# Medicinal chemistry perspective of pyrido[2,3-*d*]pyrimidines as anticancer agents

**DOI:** 10.1039/d3ra00056g

**Published:** 2023-02-28

**Authors:** Adarsh Kumar, Kuber Kumar Bhagat, Ankit Kumar Singh, Harshwardhan Singh, Tanuja Angre, Amita Verma, Habibullah Khalilullah, Mariusz Jaremko, Abdul-Hamid Emwas, Pradeep Kumar

**Affiliations:** a Department of Pharmaceutical Sciences and Natural Products, Central University of Punjab Ghudda Bathinda 151401 India pradeepyadav27@gmail.com; b Bioorganic and Medicinal Chemistry Research Laboratory, Department of Pharmaceutical Sciences, Sam Higginbottom University of Agriculture Technology and Sciences Prayagraj 211007 India; c Department of Pharmaceutical Chemistry and Pharmacognosy, Unaizah College of Pharmacy, Qassim University Unayzah 51911 Saudi Arabia h.abdulaziz@qu.edu.sa; d Smart-Health Initiative and Red Sea Research Center, Division of Biological and Environmental Sciences and Engineering, King Abdullah University of Science and Technology P.O. Box 4700 Thuwal 23955-6900 Saudi Arabia Mariusz.jaremko@kaust.edu.sa; e King Abdullah University of Science and Technology, Core Labs Thuwal 23955-6900 Saudi Arabia abdelhamid.emwas@kaust.edu.sa

## Abstract

Cancer is a major cause of deaths across the globe due to chemoresistance and lack of selective chemotherapy. Pyrido[2,3-*d*]pyrimidine is an emerging scaffold in medicinal chemistry having a broad spectrum of activities, including antitumor, antibacterial, CNS depressive, anticonvulsant, and antipyretic activities. In this study, we have covered different cancer targets, including tyrosine kinase, extracellular regulated protein kinases – ABL kinase, phosphatidylinositol-3 kinase, mammalian target of rapamycin, p38 mitogen-activated protein kinases, BCR-ABL, dihydrofolate reductase, cyclin-dependent kinase, phosphodiesterase, KRAS and fibroblast growth factor receptors, their signaling pathways, mechanism of action and structure–activity relationship of pyrido[2,3-*d*]pyrimidine derivatives as inhibitors of the above-mentioned targets. This review will represent the complete medicinal and pharmacological profile of pyrido[2,3-*d*]pyrimidines as anticancer agents, and will help scientists to design new selective, effective and safe anticancer agents.

## Abstract 2

Pyrido[2,3-*d*]pyrimidines against various types of cancer.

## Graphical abstract

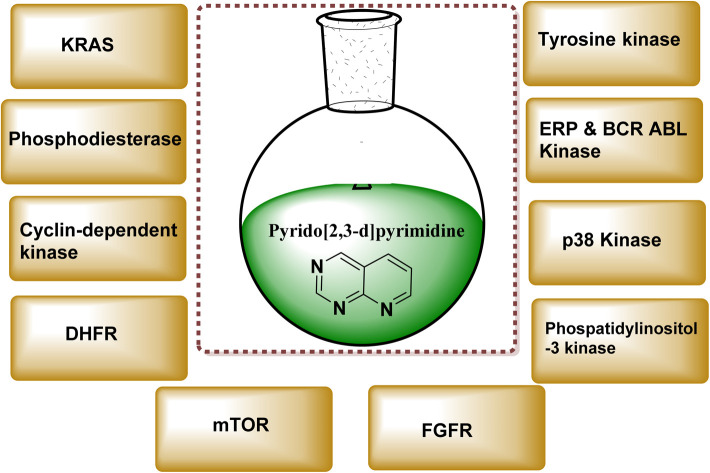

## 1 Introduction

Cancer is the explosive growth of abnormal cells that typically grow beyond their original boundaries, invade surrounding areas, spread to other organs, and result in metastasis, which is one of the main causes of cancer-related death,and second most common cause of death across the globe. Around 10.0 million cancer-related fatalities (9.9 million excluding nonmelanoma skin cancer) and 19.3 million new cancer cases (18.1 million excluding nonmelanoma skin cancer) were estimated in 2020 across the globe. The most lethal malignancies include lung (1.8 million), colorectal (935 000), liver (830 000), stomach (769 000), and breast cancer. The most frequent cancers worldwide are lung (2.2 million), breast (2.09 million), colorectal (1.9 million), prostate (1.28 million), skin (1 million), and stomach (1 million) (627 000).^1^ Cancer is the Latin term meaning crab. Because of the crab-like tenacity, a malignant tumor often appears to grab the tissues it invades, and the ancients used the term to indicate a malignancy.^2^ Cancer cells (defined by their uncontrollable growth and invasion of other tissues) do not show the same growth average as a normal heathly cell. Normal cells multiply and expand, controlled to produce additional cells as required to maintain a healthy body. Cells die as they grow old or are damaged and are replaced by new cells. Cancer cells, rather than dying, continue to proliferate and generate new, aberrant cells. Normal cells cannot invade (grow into) other tissues. The other cells can combine to form a mass of tissues known as a tumor. Not all tumors are cancerous; they might be benign (not cancerous) or malignant (cancerous).^3^ The worldwide burden of cancer is anticipated to rise significantly due to population growth, age, and increase in risk factors like smoking, inactivity, poor food, and infections that cause cancer. It is well established that cancer diagnosis and treatment have substantial and long-term consequences on both patient and caregiver physical, emotional, and spiritual well-being.^3,4^

### 1.1 Pyrido[2,3-*d*]pyrimidine

**Fig. 1:**
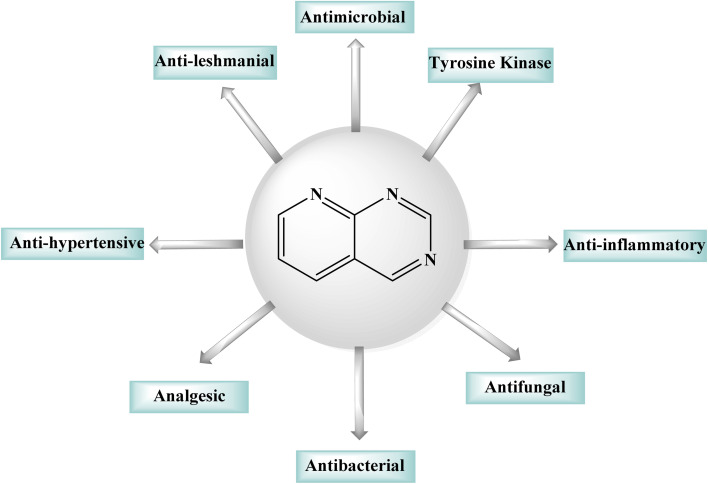
Pharmacological activities of the pyrido[2,3-*d*]pyrimidine derivatives.

Purines, quinazolines, pteridines, and pyrido-pyrimidines are examples of bicyclic nitrogen-containing heterocyclic compounds that are well-known pharmacophores in medicinal chemistry. Examples of commercial medications with a bicyclic main structure include the tyrosine kinase inhibitors gefitinib and erlotinib, and both are quinazoline derivatives. Both of them are used to manage non-small cell lung cancer. Pyrido[2,3-*d*]pyrimidines have been studied extensively as quinazoline analogs.^5,6^ Pyrido[2,3-*d*]pyrimidines have shown antitumor, antibacterial, CNS depressive, anticonvulsant, antipyretic, and analgesic effects, as shown in Fig. 1.^6^ Particularly, pyrido[2,3-*d*]pyrimidines have been shown to be effective against *Toxoplasma gondii* and *Pneumocystis carinii* (tg) culture of tumor cell lines, and the activity is attributed to dihydrofolate reductase inhibition.^7–9^ Due their diverse anticancer or antiproliferative properties, pyrido[2,3-*d*]pyrimidine derivatives were great interest of reseachers working in this field.^10,11^ Fusing a pyridine and a pyrimidine ring results in pyridopyrimidines, ortho-fused bicyclic heterocyclic structures. Pyridopyrimidines (1,3,8-triazanaphtalenes) are one of the four isomeric pyridopyrimidines.^12,13^ Because of their proximity to DNA bases, such structures are included in the favored heterocyclic scaffolds hypothesis for drug development, which was suggested by Evans in the late 1980s and recently refined by Altomare.^14,15^ Pyrido[2,3-*d*]pyrimidine is a favored heterocyclic scaffold that can act as ligands for various receptors in the body.^16^

## 2 Synthetic strategies of pyrido[2,3-*d*]pyrimidines

**Fig. 2:**
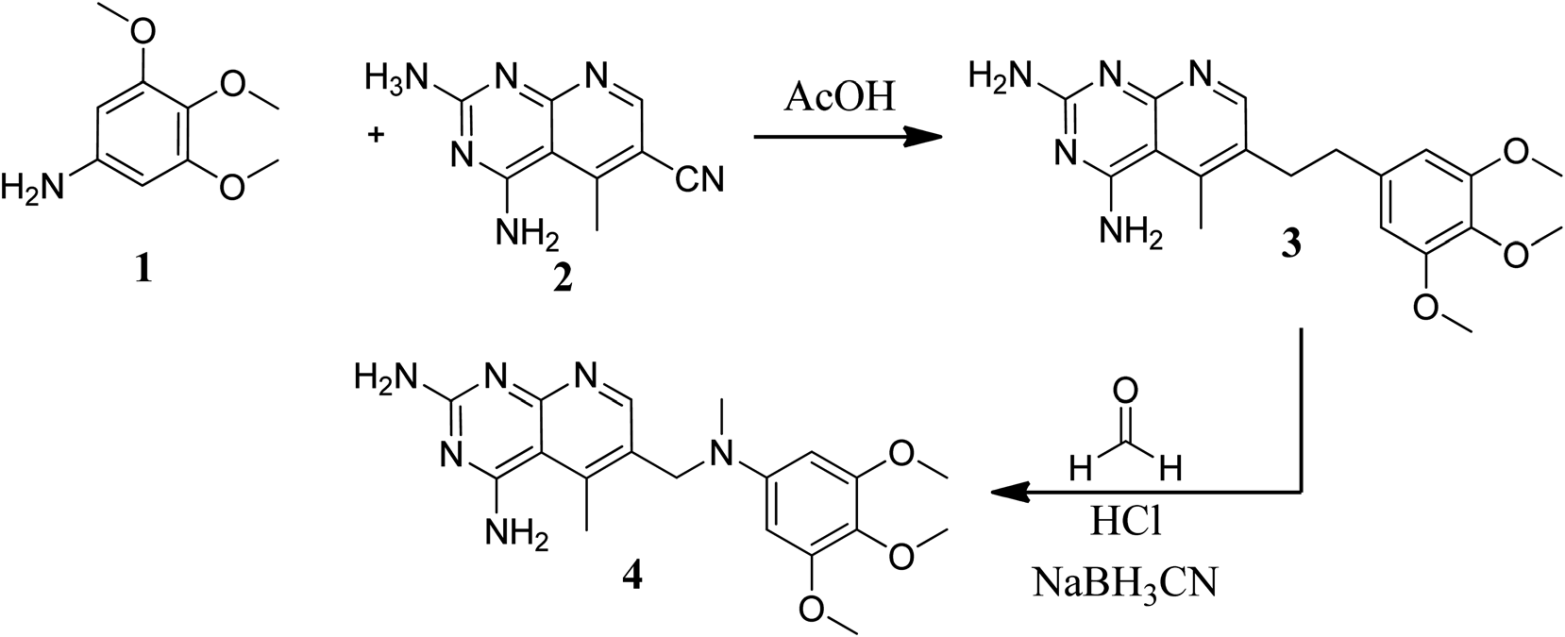
Scheme for the synthesis of pyrido[2,3-*d*]pyrimidine derivative (4).

Various methods for developing pyrido[2,3-*d*]pyrimidines have been previously reported *via* condensation techniques and pyridine annelation reactions, in solid as well as solution phase. In 1993, Kisliuk *et al.* described the synthesis of pyrido[2,3-*d*]pyrimidine-2,4-diamines as shown in Fig. 2. They synthesized precursor (3) in the presence of RANEY® Ni (70%) in acetic acid by deductive condensation of 6-cyano-5-methyl-pyrido[2,3-*d*]pyrimidine-2,4-diamine (2) with 3,4,5-trimethoxyaniline (1), which was then methylated at the N_10_ position by reductive alkylation with formaldehyde and sodium cyanoborohydride.^17,18^

**Fig. 3:**
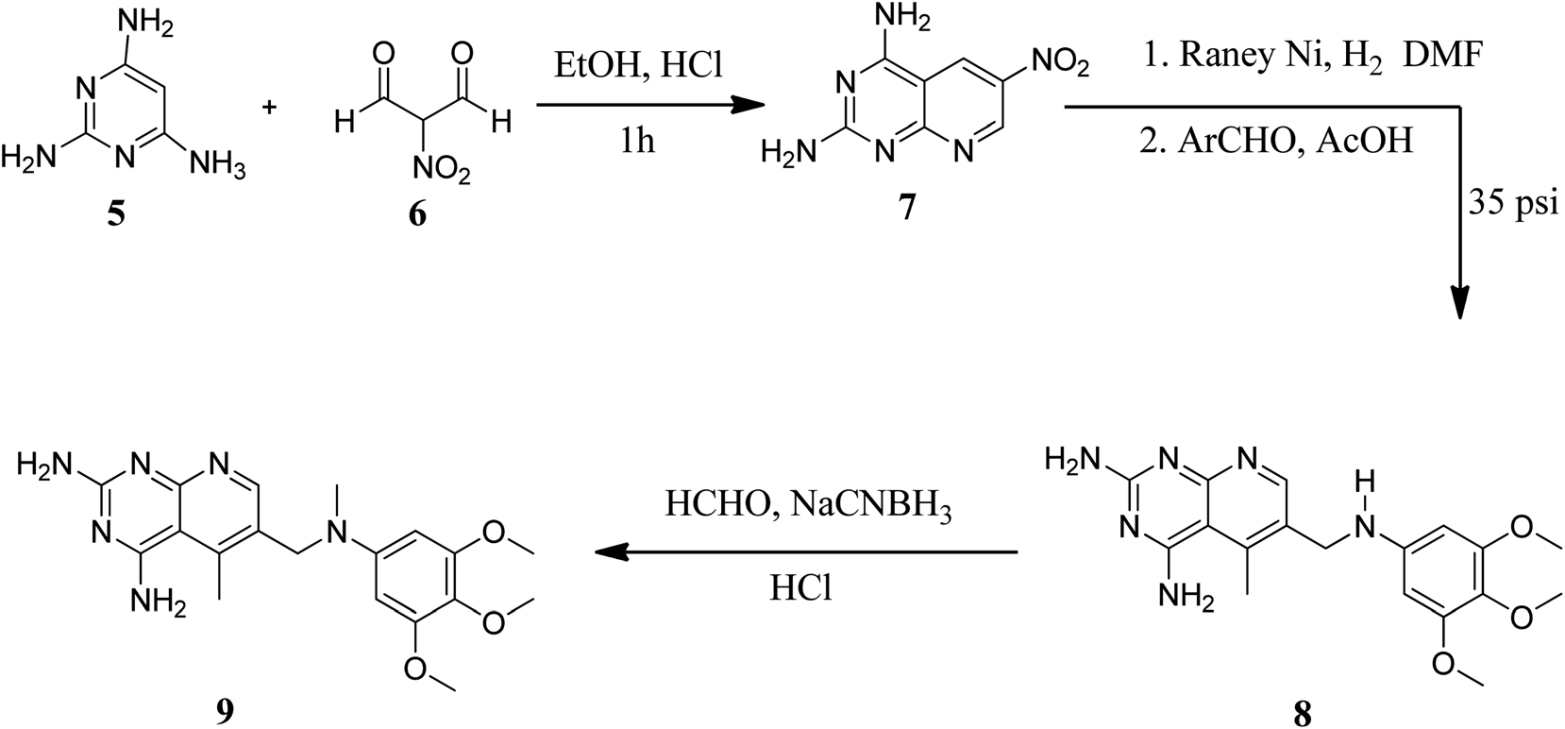
Scheme for the synthesis of pyrido[2,3-*d*]pyrimidine diamine derivative (9).

They also created a different method to synthesize pyrido[2,3-*d*]pyrimidine-2,4-diamines (9) by mixing 2,4,6-triaminopyrimidine (5) with the sodium salt of nitromalonaldehyde and obtained create the 2,4-diamino6-nitropyrido[2,3-*d*]pyrimidine (7) in a single step. This compound was subsequently reduced to its 6-amino homolog by RANEY® Ni using DMF. By using several aldehydes in reductive amination, the desired product 8 was produced (ArCHO = 3,4,5-trimethoxybenzaldehyde). In the final step, formaldehyde was used to *N*-methylate 8 in the presence of sodium cyanoborohydride (Fig. 3).^18,19^

**Fig. 4:**
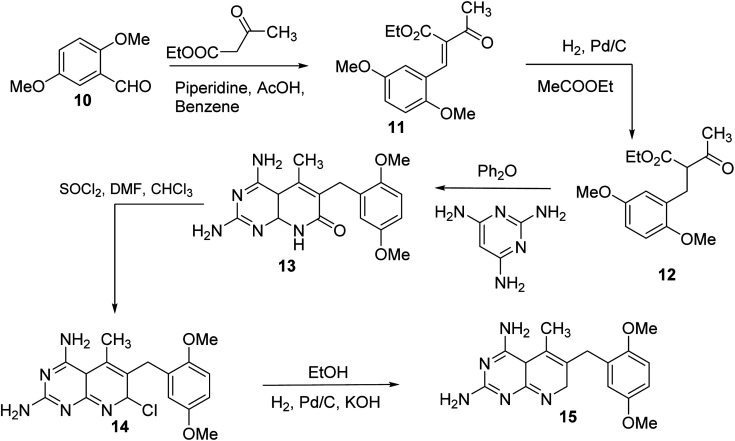
Scheme for the synthesis of pyrido[2,3-*d*]pyrimidine derivative (15).

N6-[(2,5-dimethoxyphenyl)methyl]-N6-methylpyrido[2,3-*d*]pyrimidine was produced by Queener *et al.* In order to get the necessary 2,4,6-triaminoquinazoline, the 2,4-diamino-6-nitroquinazoline (7) was reduced with hydrogen and RANEY® nickel around 30–35 psi (10). Then, 2,5-dimethoxybenzaldehyde (ArCHO) was added to produce the N_9_–H precursor (11). Using sodium cyanoborohydride, the reductive N_9_-alkylation process was used to produce the end product. Queener *et al.* also investigated the biological effects of compound 12 as a lipophilic inhibitor of dihydrofolate reductase.^20^ Piritrexim was first synthesized by Grivsky *et al.* possessed anti-parasitic, anti-folate, anti-psoriatic, and anti-tumor characteristics. Piritrexim suppressed dihydrofolate reductase (DHFR) and had anticancer effects in rats with carcinosarcoma. Compared to certain analogs, Piritrexim has the advantage of not acting as a histamine metabolism inhibitor, reducing the danger of adverse metabolic effects.^21^ In the presence of a combination of piperidine and glacial acetic acid, 2,5-dimethoxybenzaldehyde (10) was reacted with ethyl acetoacetate in refluxing benzene to produce ethyl-acetyl-(2,5-dimethoxyphenyl) acrylate (11). The latter was subsequently hydrogenated with 5% Pd/C (catalyst) to produce the required ethyl-acetyl-(2,5-dimethoxyphenyl) propionate (12). Then, 12 was condensed using 2,4,6-triaminopyrimidine in diphenyl ether at 195–230 °C. 2,4-Diamino-7,8-dihydro-6-(2,5-dimethoxybenzyl)-5-methyl -7-oxopyrido[2,3-*d*]pyrimidine (13) was treated with a 1 : 1 combination of *N*,*N*-dimethylformamide thionyl chloride to produced -5-methylpyrido[2,3-*d*]pyrimidine2,4-diamine (14). The hydrogenolysis of 14 with Pd/C in the presence of potassium hydroxide produced the desired PTX (15) (Fig. 4).^18,21^

**Fig. 5:**
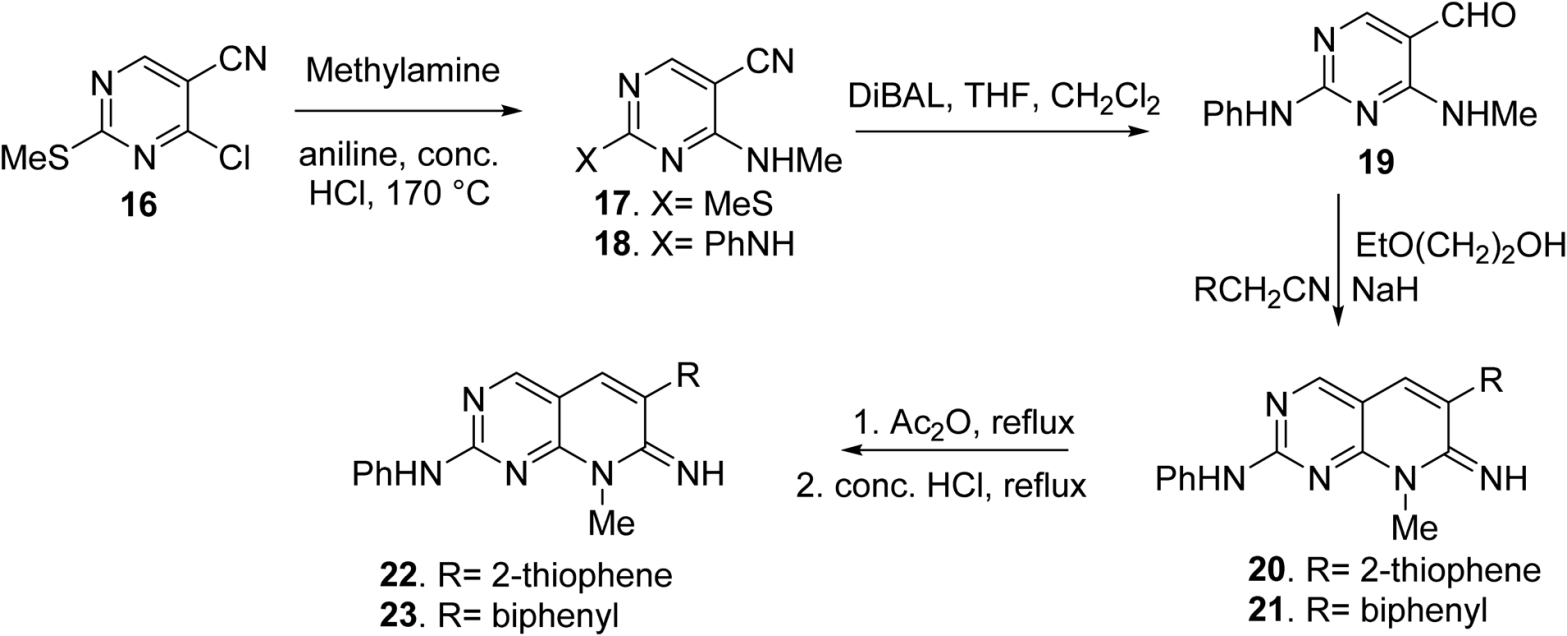
Scheme for the synthesis of pyrido[2,3-*d*]pyrimidine derivatives.

Blankley *et al.* synthesized 8-methyl-2-phenylamino-6-thiophene-2-yl-8*H*-pyrido-[2,3-*d*]pyrimidin-7-one (22) and 6-(biphenyl-4-yl)-8-methyl-2-phenylamino-8*H*-pyrido-[2,3-*d*]pyrimidin-7-one (23) by treating 4-chloro-5-cyano2-methylsulfanyl-pyrimidine (16) with methylamine (17). In the presence of a catalytic quantity of strong hydrochloric acid, the 2-methyl sulphide group of 17 was replaced with aniline, yielding compound 18. The aldehyde (19) was obtained by reducing the nitrile group of 18 with diisobutylaluminum hydride and then hydrolyzing it. The condensation of 19 with 2-thiopheneacetonitrile or 4-biphenylacetonitrile with 2-ethoxyethanol and sodium hydride, respectively, yielded the pyridopyrimidin-7-imines 20 and 21. Compounds 22 and 23 were obtained *via* acetylation of the imines followed by acid hydrolysis (Fig. 5).

**Fig. 6:**
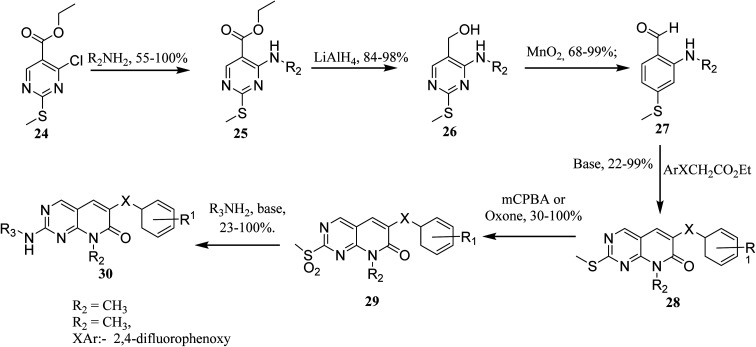
Scheme for the synthesis of pyrido[2,3-*d*]pyrimidine derivatives.

In order to synthesize intermediate esters (25), ethyl 4-chloro-2-methylthiopyrimidine-5-carboxylate (24) was combined with several amines. Alcohols (26) were then converted to 4-aminopyrimidine-5-carboxaldehydes through oxidation (27). To make the core pyrimidinopyridones, aldehydes (27) were condensed with appropriately substituted acetates. In order to synthesize the desired molecules (30), the methylthio groups of pyrimidinopyridones (28) were oxidized to produce the representative sulfones (29). The most frequent path was needed for 8-substituent chains since they were more intricate than straight chains (Fig. 6)^22^

**Fig. 7:**
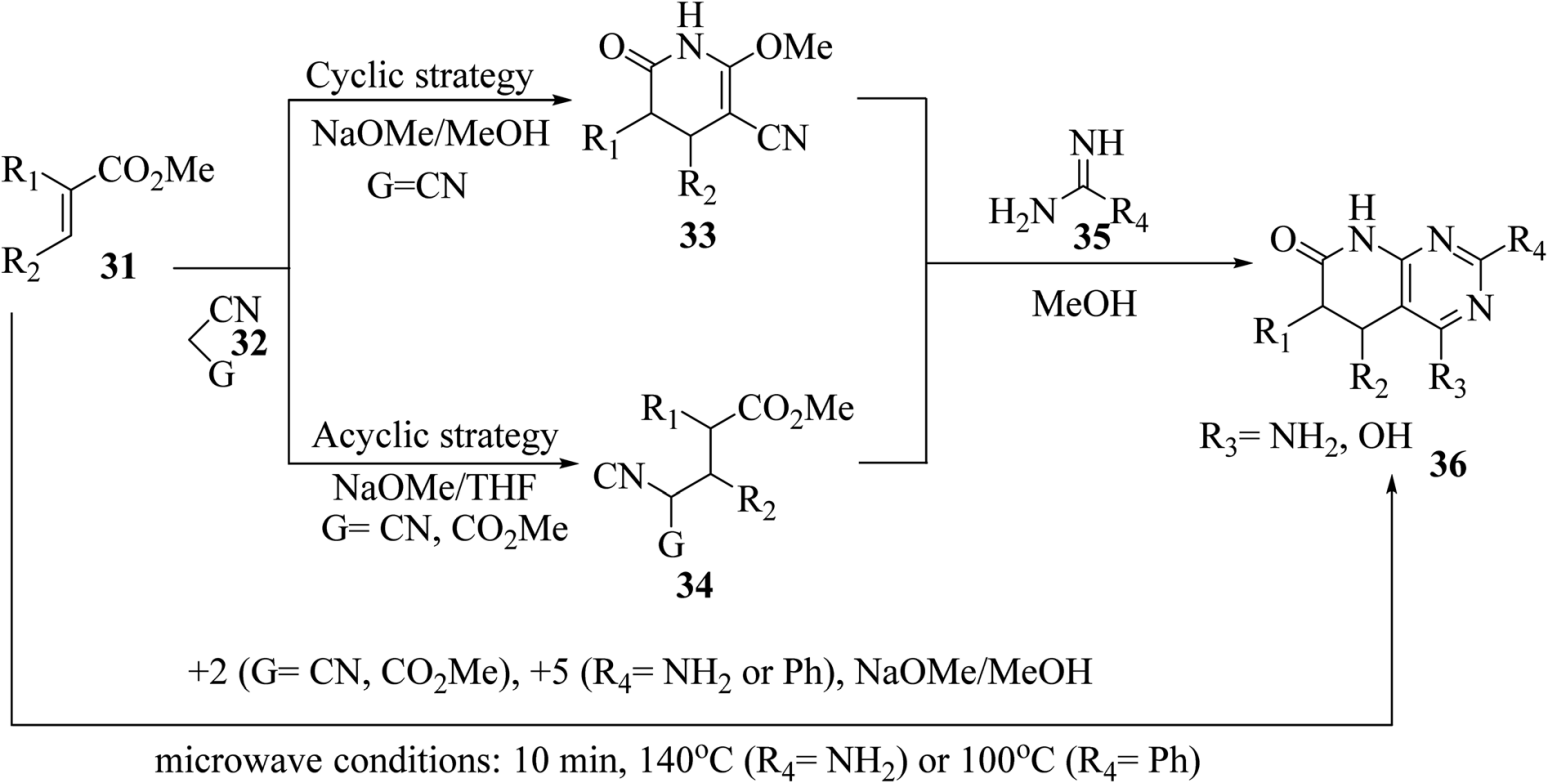
Scheme for the synthesis of pyrido[2,3-*d*]pyrimidine derivatives.

The synthesis of the reduced form of the target heterocycle in a highly regioselective manner has been reported using 6-aminouracil derivatives as the enamine component in a Michael or Hantzsch-type condensation. This strategy has several advantages over current methods, including using readily available alkynones as Michael acceptors, effectively eliminating the need for subsequent oxidation, and providing pyrido[2,3-*d*]pyrimidine derivatives directly without purification and with total regiochemical control.^23^ Victory and colleagues investigated a heterocyclic system that can be produced in a multistep sequence using 2-methoxy-6-oxo-1,4,5,6-tetra-hydropyridin-3-carbonitrile intermediates [created by reacting an α,β-unsaturated ester (31) with malononitrile (32, G = CN) in MeOH/NaOMe]. Because pyridones 33 include a highly reactive methoxy group, they are suitable substrates for a subsequent nucleophilic substitution or condensation processes. By treating pyridones 33 with amidine systems 35 (R_4_ = NH_2_, H, Me, Ph), they have created extensive procedures for the synthesis of bicyclic heterocycles such as pyrazolo[3,4-*b*]pyridines, 1,6-naphthyridines, and 4-amino-pyrido[2,3-*d*]pyrimidines 36 (R_3_ = NH_2_). In another study, they described an acyclic variation of the protocol for synthesizing pyridopyrimidines 36 (R_3_ = NH_2_), focusing on isolating the corresponding Michael adduct 34 (*G* = CN). This allowed them to also produce 4-oxopyrido[2,3-*d*]pyrimidines 36 (R_3_ = OH) by treating intermediates 34 (*G* = CO_2_Me), which were produced by the Michael addition of acrylate (31) and methyl cyanoacetate (32, G = CO_2_Me), with an amide analog (35). They showed that pyrido[2,3-*d*]pyrimidines 36 may be synthesized in a single step using a microwave-assisted cyclocondensation of α,β-unsaturated ester, amidine, with malononitrile/cyanoacetate (Fig. 7).^24^

**Fig. 8:**
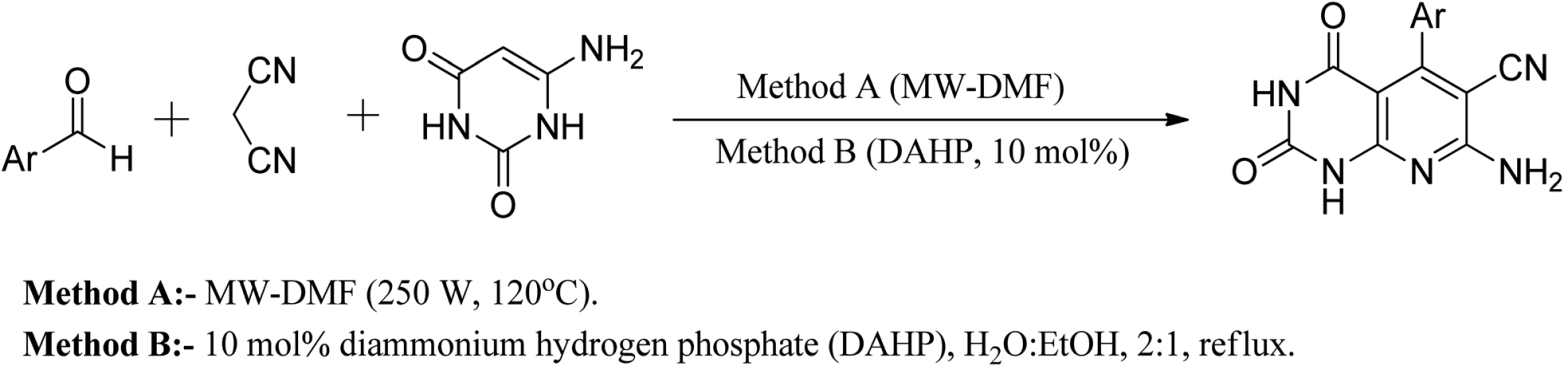
General method of synthesizing pyrido[2,3-*d*]pyrimidine derivatives.

Another approach reported in the literature is using nanocrystalline MgO in the reaction of 6-aminouracil, 6-amino-2-thiouracil, or 6-amino-1,3-dimethyluracil with malononitrile and aldehydes in water at 80 °C to produce a series of pyrido[2,3-*d*]pyrimidine derivatives in high yields.^25^ In a different strategy, they demonstrated an efficient one-pot three-component reaction of aromatic aldehydes, malononitrile, and (6)-aminouracil and MWI for the synthesis of pyrido[2,3-*d*]pyrimidines using microwave irradiation (method A) and also in the presence of catalytic amounts of DAHP (10 mol percent) aqueous ethanol under reflux (method B) (Fig. 8).^26^

**Fig. 9:**
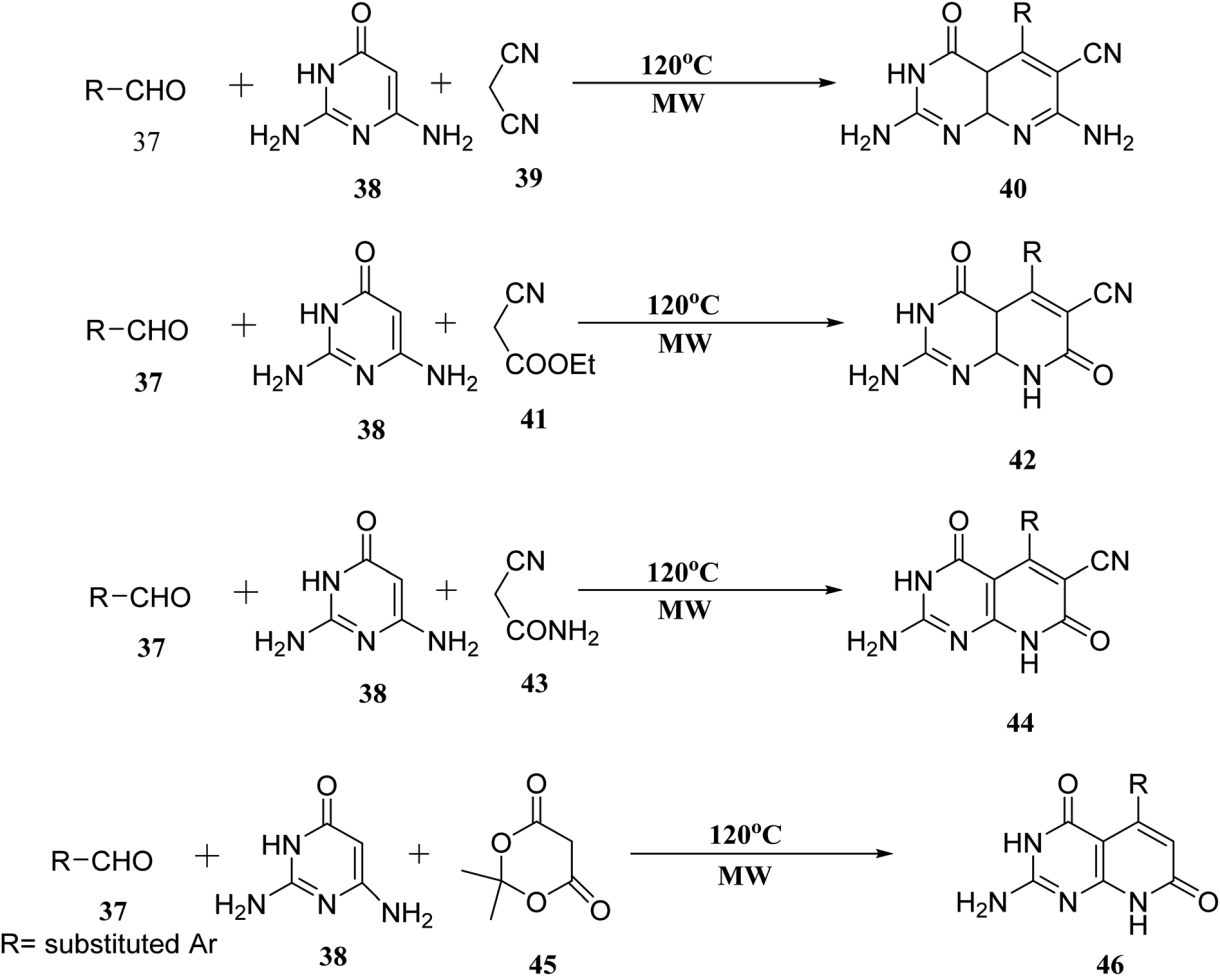
Scheme for the synthesis of pyrido[2,3-*d*]pyrimidine derivatives.

When the reaction of aldehyde, 2,6-diaminopyrimidin-4-one (38), and malononitrile (39) was performed in the presence of glycol under microwave irradiation, the derivative (40) was generated in good yields. Aldehyde (37), 2,6-diaminopyrimidin-4-one (38), and ethyl cyanoacetate (41) were carried out under the same conditions. When 2-cyanoacetamide was used in place of ethyl cyanoacetate (41), dehydrogenation of (42) was also accomplished (43). Meldrum's acid (45), (37), and (38), used as starting materials for microwave irradiation, produced the required compounds (46) in significant amounts (Fig. 9).^27^

## 3 Molecular targets of pyrido[2,3-*d*]pyrimidine derivatives

Many antitumor activities have been reported for pyrido[2,3-*d*]pyrimidines, which could be attributable to the inhibition of various enzymes involved in carcinogenesis.^9^

### 3.1 Tyrosine kinase

Tyrosine kinases are essential components of the signaling cascade that controls cell division, metabolism, migration, and the death of cells. Tyrosine kinases use ATP to catalyze the phosphorylation of particular tyrosine residues in their protein targets.^28^ Tyrosine kinases play an essential role in several stages of the emergence and spread of cancer. Tyrosine kinase signal transduction typically regulates excessive proliferation or increases apoptotic sensitivity. These signaling pathways are frequently genetically or epigenetically changed in cancer cells to provide specific benefits to cancer cells. As a result, it is no surprise that abnormally increased signaling from tyrosine kinases gives these enzymes a dominant oncoprotein status, resulting in signaling network failure.^29^

#### 3.1.1 Biological pathway and inhibition of tyrosine kinase

**Fig. 10:**
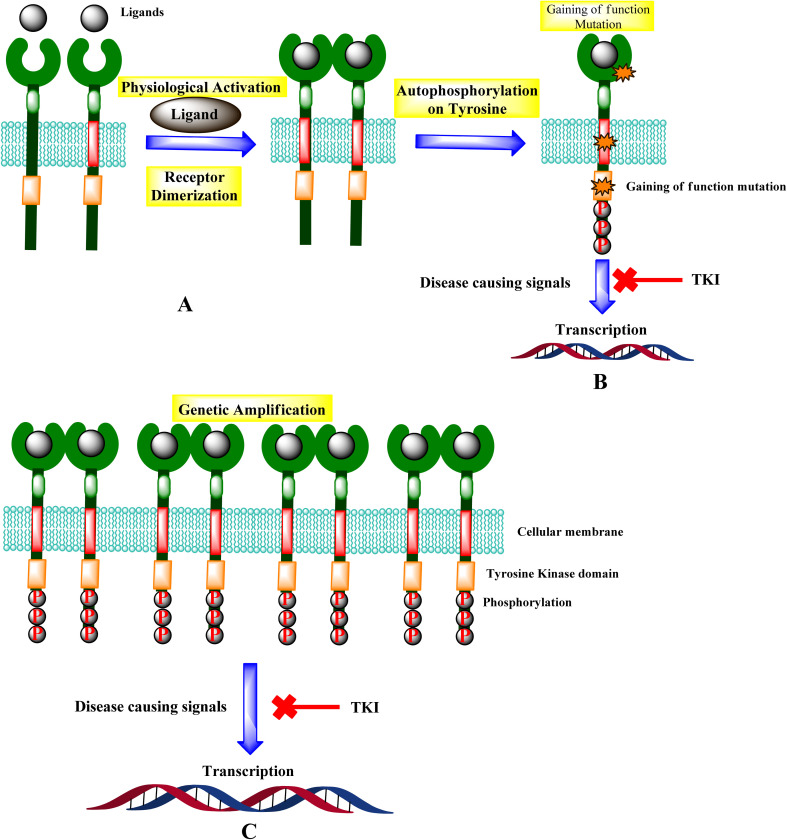
Mechanisms of RTK activation. (A) RTK activation. (B) Potential gain-of-function mutations and the signal inhibition. (C) Genetic amplification leads to the transcription, and TKI leads to the inhibition of diseases causing signals.

Receptor tyrosine kinase (RTKs) are usually activated by ligands that target specific receptors. Growth factor ligands bind to RTK's extracellular regions, and the receptor activation is carried out by ligand-induced dimerization or oligomerization.^30^ Ligand attached to the extracellular domain stabilizes the generation of active dimers, which activates protein tyrosine kinases. The concentration of RTKs is increased by receptor oligomerization, enabling effective transphosphorylation of tyrosine residues inside the activation loop of the catalytic domain. For most RTKs, the resulting conformational alterations allow *trans*-autophosphorylation of each TKD and the release of *cis*-autoinhibition (Fig. 10).^31,32^

Genetic amplification is the principal mechanism that leads to the overexpression of RTKs and transcription enhancement.^33,34^ SH2 domain proteins interact with phosphorylated receptors and become phosphorylated as well. The growth factor Ras/Raf/mitogen-activated protein map phosphorylated cascade then connects with the substance of the g-protein system, allowing the cell to migrate and express itself.^30,35^

#### 3.1.2 Mechanism of action of tyrosine kinase inhibitors

**Fig. 11:**
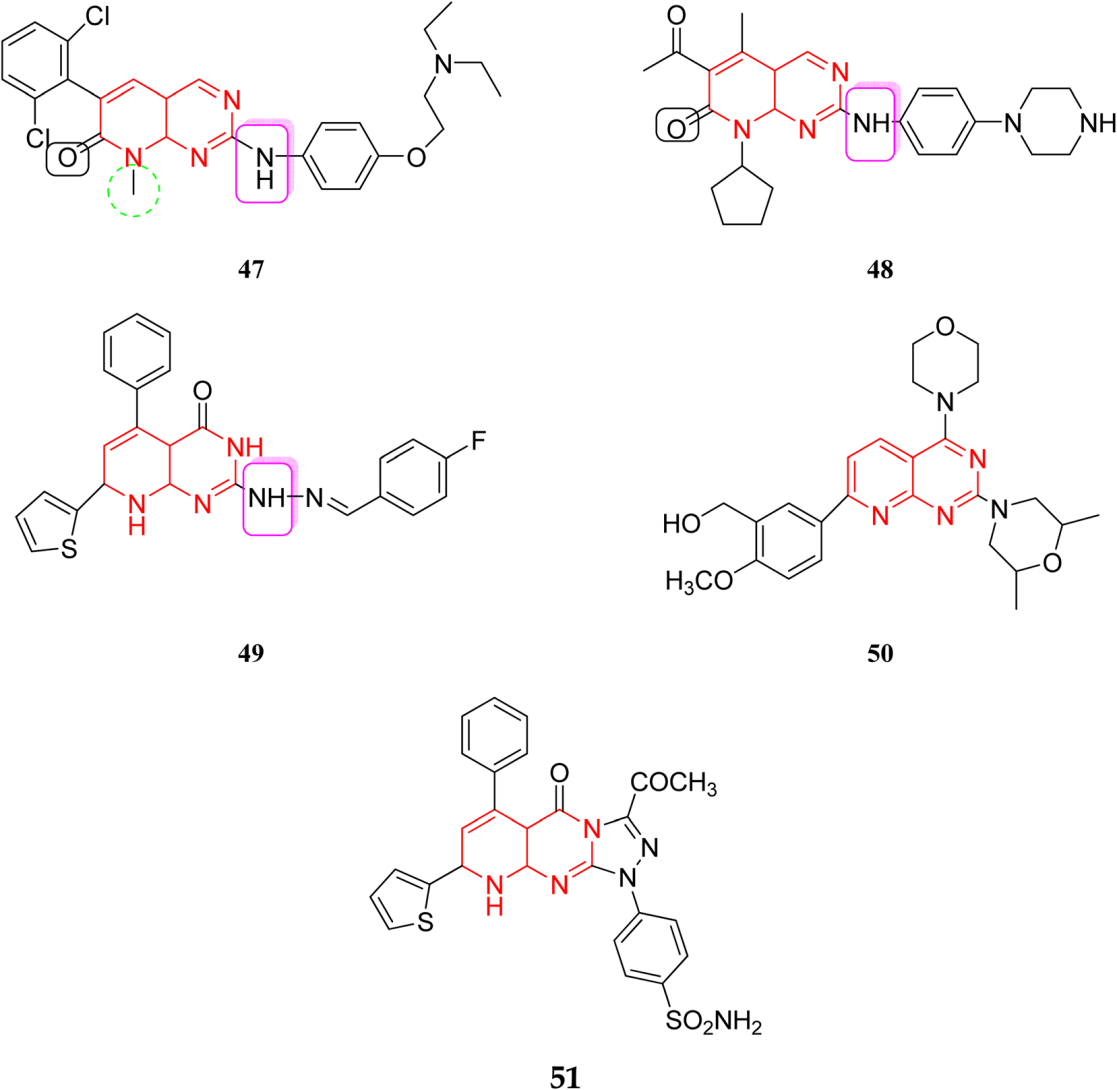
Reported pyrido[2,3-*d*]pyrimidine as kinase inhibitors.

Several amino acids of substrate enzymes are phosphorylated by tyrosine kinases, leading to different changes in the downstream cellular biology and signaling. TKs can alter cell growth, migration, differentiation, apoptosis, and death by triggering downstream signal transduction. Constitutive activation or inhibition, whether caused by mutations or other methods, can result in dysregulated signal cascades, which can lead to cancer and other diseases.^35,36^ The tyrosine kinase inhibitors inhibit this dysregulated signal cascade and block transcription in both genetic amplification and mutation, leading to the disease-causing signals.^36^ The compounds 47–51 showed excellent activity against various kinases, including TKs, PI3K, and CDK4/6. In molecule 47, the presence of dichloro and tertiary amine increased the activity. In molecule 48, piperazine with amine linkage and acetyl group at pyridine ring increased the activity, thiophene, phenyl with fluorine atom were responsible for activity in 49, presence of methoxy and morpholine were necessary for activity in molecule 50, also other triazole rings in 51 enhanced the activity (Fig. 11).^37–40^

In 2018, Elzahabi *et al.* successfully synthesized substituted pyrido[2,3-*d*]pyrimidines and tested their ability to inhibit the growth of five cancer cell lines. Pyrido[2,3-*d*]pyrimidines, were found to have significant inhibitory effects against a variety of kinases, including TKs, PI3K, and CDK4/6.^9^

#### 3.1.3 Structure activity relationship of tyrosine kinase inhibitors

**Fig. 12:**
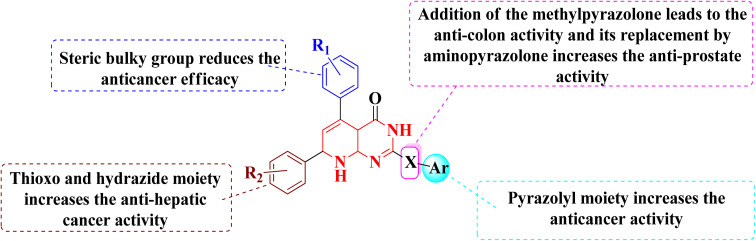
SAR of pyrido[2,3-*d*]pyrimidine derivatives.

**Fig. 13:**
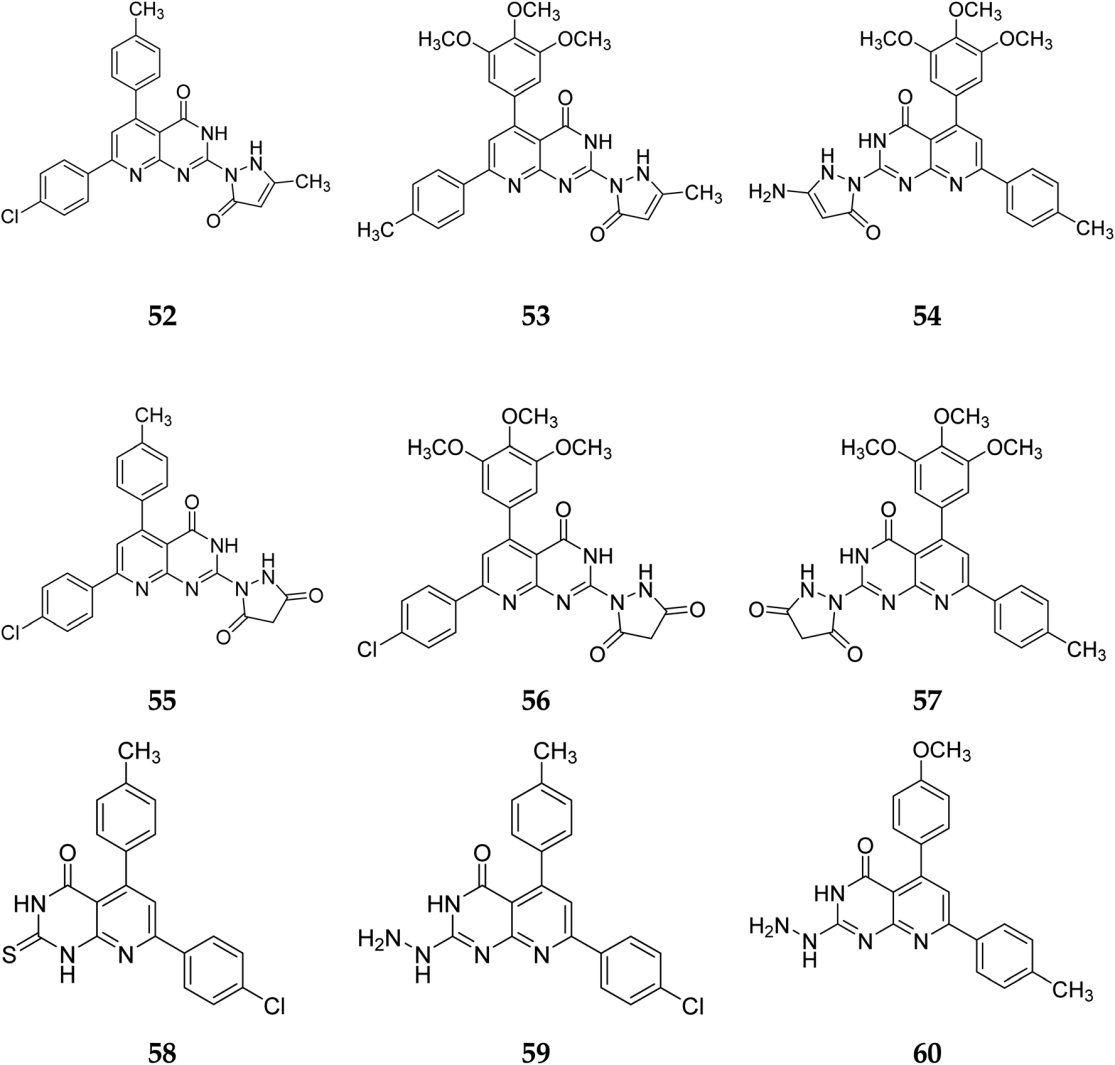
Pyrido[2,3-*d*]pyrimidine derivatives reported as tyrosine kinase inhibitors.

According to the structure–activity relationships of the screened products, presence of carbonyl at C-2 of pyrido[2,3-*d*]pyrimidine derivatives (52–56) provided the maximum anticancer activity. Particularly 52 and 53, which had (4-CH_3_-phenyl) and (4-chlorophenyl) at C-5 and C-7, respectively, shared the same pyridopyrimidine scaffold. Compound 53 containing 3-methyl-5-oxopyrazolyl moiety of showed strong anticancer efficacy against prostate, colon, and liver malignancies. The anticancer action was switched from anti-colon to anti-prostate cancer when the methylpyrazolone moiety in 53 was replaced with aminopyrazolone in 54 (Fig. 12 and 13).

In the same way, adding a steric bulky group to 56 and 57 reduced its anticancer efficacy. Converting the thioxo group in 58 to the hydrazide moiety in 59 significantly enhanced the anti-hepatic cancer activity. The electronic component was able to have a positive impact on anticancer activity due to the hydrophilic electron-rich nature of the hydrazide molecule.^9^

A novel class of substituted pyrido[2,3-*d*]pyrimidinones was investigated for their *in vitro* anticancer efficacy. Compounds 52 and 55 were more effective (IC_50_ = 0.3 μM) than doxorubicin in the HepG-2 cell line. In addition, compound 59 was approximately equivalent to doxorubicin (IC_50_ = 0.6 μM). Compounds 60 and 52 were having higher activity than doxorubicin (IC_50_ = 5.47, 6.6, and 6.8 μM, respectively) in a cellular screening on PC-3. The activity of 60, 52, and 53 was two-fold as that of doxorubicin in the HCT-116 cell line (IC_50_ of 6.9, 7, 5.9 μM, and 12.8 μM, respectively). Synthesized compounds showed significant growth inhibitory effects in HepG-2, HCT-116 as well as PC-3 cell lines with reference to doxorubicin, but on MCF-7 and A-549 cancer cells showed comparetively low activity. PDGFR β, EGFR, and CDK4/Cyclin D1 kinases were all inhibited by the potent anticancer molecule 52 (Fig. 13).^9^

**Fig. 14:**
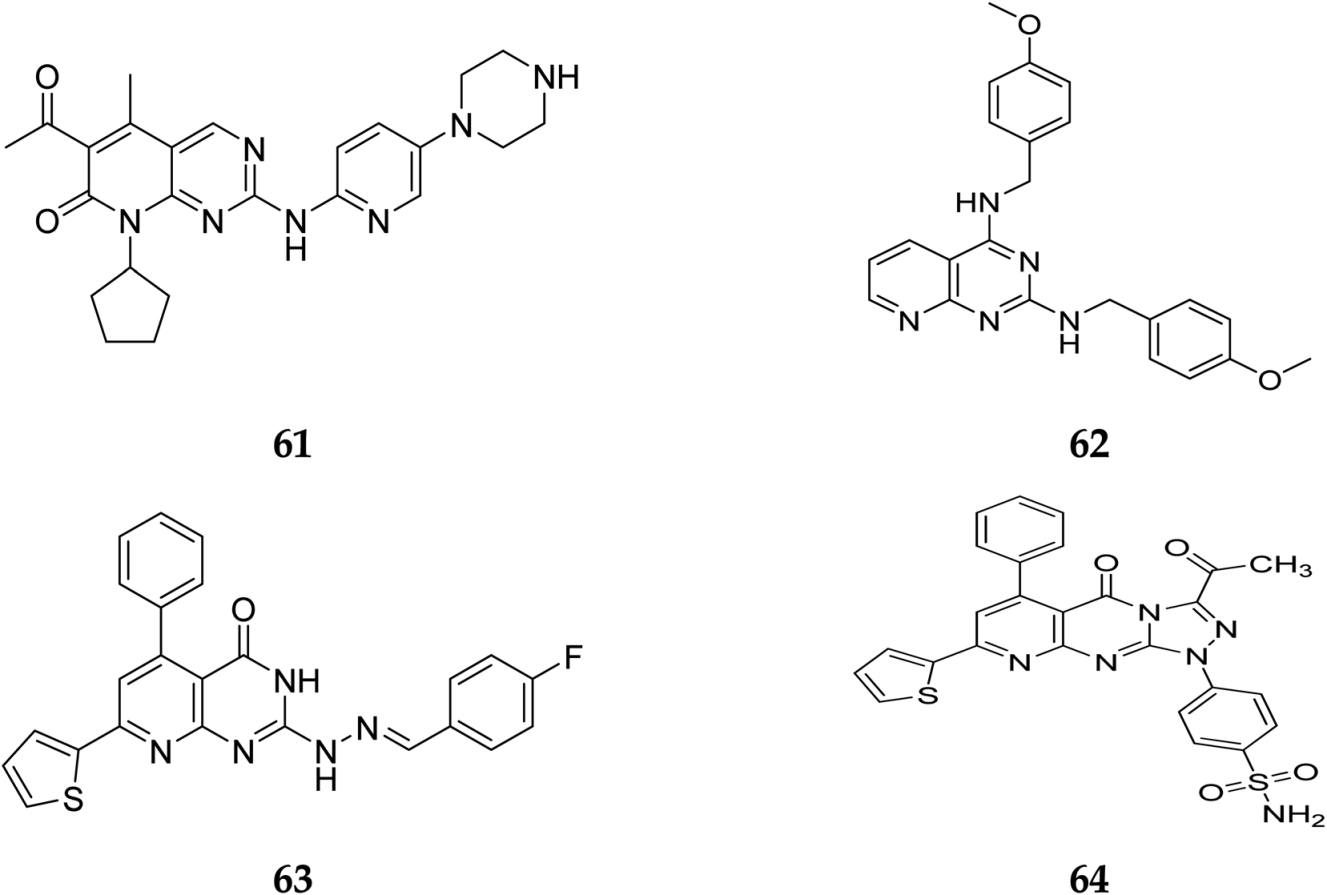
Pyrido[2,3-*d*]pyrimidine derivatives reported as anticancer agents.

Pyrido[2,3-*d*]pyrimidine is a vital core known to play a role in many active compounds with diverse activities, particularly chemotherapeutic, which may be capable of inhibiting cyclin-dependent kinase (CDK). For example, The US Food and Drug Administration has authorized the CDK4/6 inhibitor palbociclib (61), which is used to treat breast cancer. In addition, 2,4-diaminopyrido[2,3-*d*]pyrimidine (62) is a promising anticancer drug with dose-dependent antiangiogenic and proapoptotic actions through induction of 2 separate signaling pathways, notably G2/M cell cycle arrest as well as induction of apoptosis by encouraging caspase-3 activation and DNA fragmentation. Furthermore, compound 63 showed remarkable anticancer activity against prostate (PC-3) and lung (A-549) cancer cells with IC_50_ values of 1.54 and 3.36 μM, respectively. Furthermore, at submicromolar concentrations (0.36, 0.41 μM, respectively), the fused triazolopyridopyrimidine (64) showed greater efficacy against both cell lines, causing cell cycle arrest by increasing the cell cycle inhibitor p21 and cancer cell death by activating caspase-3 (Fig. 14).^41^

**Fig. 15:**
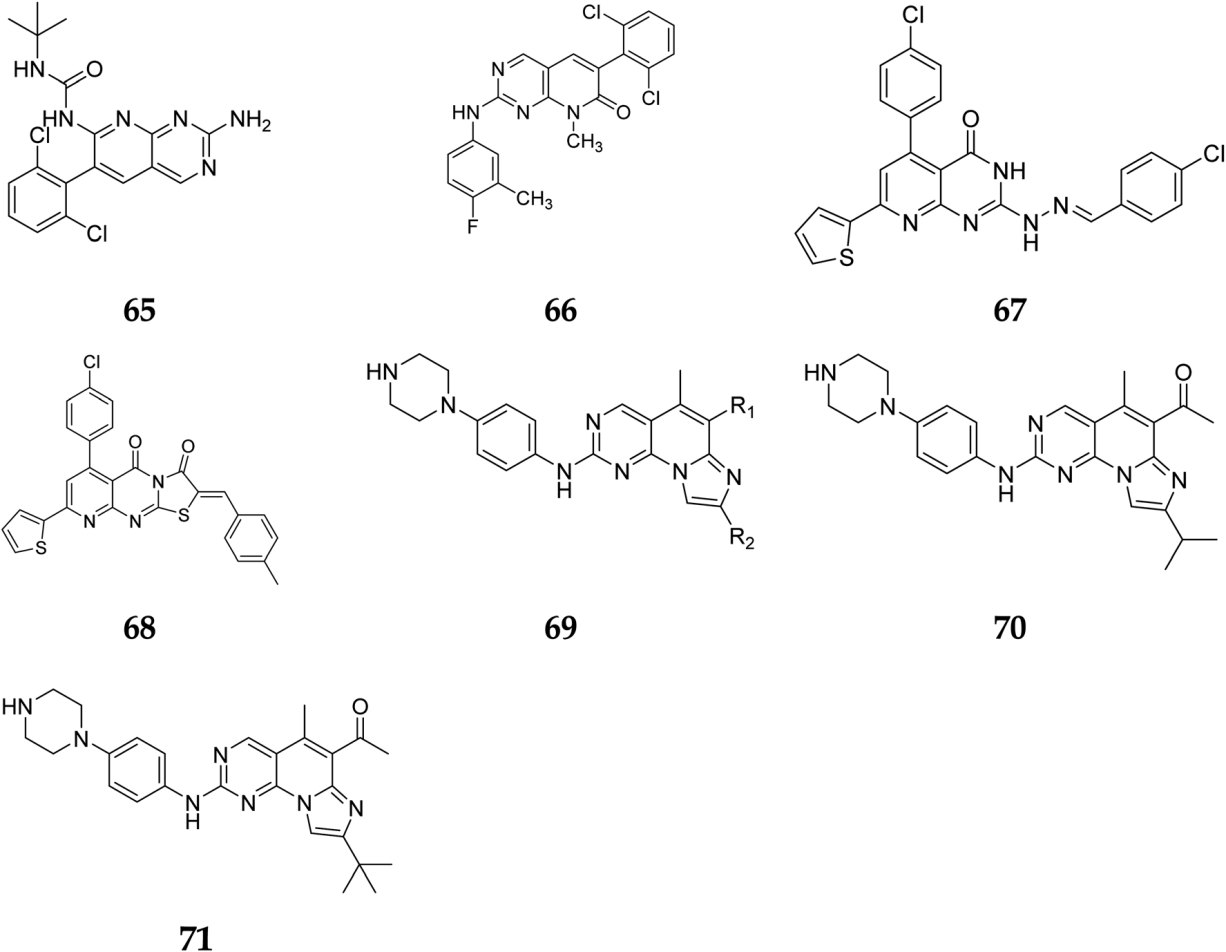
Pyrido[2,3-*d*]pyrimidine derivatives reported as anticancer agents.

Another study discovered a highly potent tyrosine kinase inhibitor (65) having IC_50_ values of 1.11, 0.13, 0.45, and 0.22 μM against the tyrosine kinases, PDGFr, FGFr, EGFr, and c-src respectively.^42^ PD180970, the derivative of pyrido[2,3-*d*]pyrimidine, acted by inhibiting ATP-competitive protein tyrosine kinase. Dorsey *et al.* found that in human K562 chronic myelogenous leukemic cells, 66 reduced *in vivo* tyrosine phosphorylation of p210Bcr-Abl (IC_50_ = 170 nM), the p210Bcr-Abl substrates Gab2 and CrkL (IC_50_ = 80 nM).^43^ Compounds 65 and 66, with IC_50_ values of 115.38 nM and 726.25 nM, were the most effective direct CDK6 inhibitors in the series. Compound 65 caused apoptosis 1.9-fold in PC-3 cells and 1.8-fold in MCF-7 cells in the apoptotic assay. The molecule 65 triggered apoptosis primarily by activating caspase-3. Compound 66, on the other hand, promoted apoptosis primarily through the intrinsic route, with direct inhibition of CDK6.^44^ Shi *et al.* proposed a new class of CDK4/6 inhibitors: imidazo[1′,2′:1,6]pyrido(2,3-*d*)pyrimidine analogs.^44,45^ In the Colo-205 as well as U87MG cell lines, CDK4/6 was found to be effectively suppressed by compounds 70 and 71. The equipotent of palbociclib and abemaciclib, compounds 70 and 71 significantly inhibited CDK4/D3 (IC_50_ = 0.8 nM and 2.7 nM, respectively) and CDK6/D3 (IC_50_ = 2.0 nM and 4.8 nM, respectively). The acetyl and isopropyl moieties at C_6_ and C_8_ positions (compound 70) represented 2.5 more possible biological activities for CDK4/cyclinD3 than CDK6/cyclinD3.^45^ Changes to the functional group *tert*-butyl at the C_8_-substituent (compound 71) did not affect CDK4/6 targeting bioactivity. Compounds 70 and 71 showed favorable antiproliferative effects, remarkable metabolic properties, useful pharmacokinetic features, and significant tumour growth inhibitions with manageable toxicity in Colo-205 and U87MG xenograft models (Fig. 15).^45^

### 3.2 Extracellular signal-regulated kinase and Phospatidylinositol-3 kinaseα

#### 3.2.1 Extracellular regulated protein kinases (ERK)

A serine/threonine protein kinase known as ERK1/2 (extracellular signal-regulated kinase) is frequently found in eukaryotic cells. It belongs to the family of MAPK (mitogen-activated protein kinases). The transfer of signals from surface receptors to the nucleus is significantly aided by ERK1/2. Apoptosis, cell proliferation, differentiation, and other activities are regulated by activated ERK1/2, which phosphorylates substrates in the cytoplasm or nucleus and causes the production or activation of particular proteins.^46^

#### 3.2.2 Phospatidylinositol-3 kinase

Many cancers, including breast, gastric, ovarian, colorectal, prostate, glioma, and endometrial cancers, have altered or amplified the activity of the critical intracellular signalling system known as PI3K (phospatidylinositol-3 kinase, or PI3K). PI3K signalling is a viable therapeutic target since it is crucial for cancer cell survival, angiogenesis, and metastasis. Clinical trials employing PI3K inhibitors are continuing and have already been completed to identify effective PI3K inhibitors that could overcome resistance to existing treatments.^47^ Critical physiological and pathological processes like metabolism, cell development, proliferation, angiogenesis, and metastasis are influenced by PI3K/AKT signalling and contribute to the progression of tumours.^48,49^ PI3K proteins are categorized into three major classes based on the structural characteristics of the proteins as well as the peculiarities of the substrates (I, II, and III). Class, I PI3Ks are classified into subtypes A and B based on how they are regulated. Class IA PI3Ks combine their regulatory (p85, p85, p55, p55, and p50) and catalytic (p110, p110, p110) subunits to form dimers.^50^

#### 3.2.3 ERK2 and PI3Kα

The combined inhibition of the MAPK and PI3K signalling pathways has been identified as a viable cancer therapy that effectively overcomes the drug resistance of MAPK signalling pathway inhibitors.^51^

**Fig. 16:**
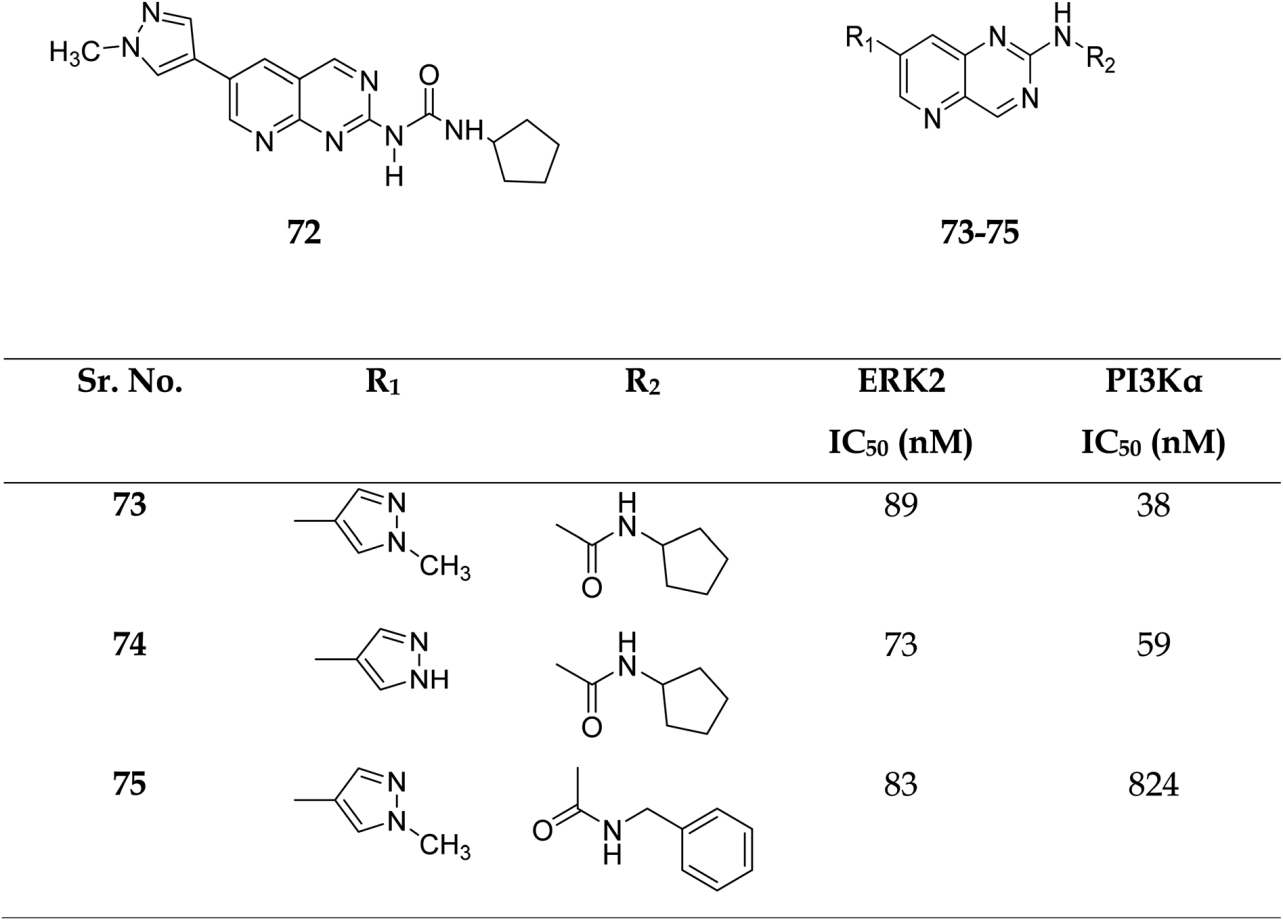
Pyrido[2,3-*d*]pyrimidine and pyrido[3,2-*d*]pyrimidine derivatives as dual inhibitors of ERK2 and PI3Kα.

Zhang *et al.* reported the scaffold-hopping synthesis of ERK/PI3K dual inhibitors by replacing 1*H*-pyrazolo[3,4-*d*]pyrimidine scaffold with pyrido[3,2-*d*]pyrimidine. Compound 73 with pyrido[3,2-*d*]pyrimidine scaffold exhibited suitable inhibitory activities on ERK2 and PI3Kα, while 72 with pyrido[2,3-*d*]pyrimidine scaffold had 1.8 and 15.1% inhibitory activity at the concentration of 1 μM on ERK2 and PI3Kα respectively (Fig. 16).^51^

Compounds 73–75 were foundd to be dual inhibitors of ERK2 and PI3K-α. Compound 74, a potent and very effective ERK and PI3K dual inhibitor, was discovered during preliminary SAR study. Compound 74 had modest ERK and PI3K inhibitory and anti-proliferative properties. Although 74 had only acceptable pharmacokinetic characteristics in SD rats, with a moderate half-life (*t*_1/2_ = 2.32 h) after intravenous treatment, it displayed significant anticancer activity *in vivo* in an HCT-116 xenograft model without eliciting obvious adverse consequences.^51^

### 3.3 Antitumor activity

**Fig. 17:**
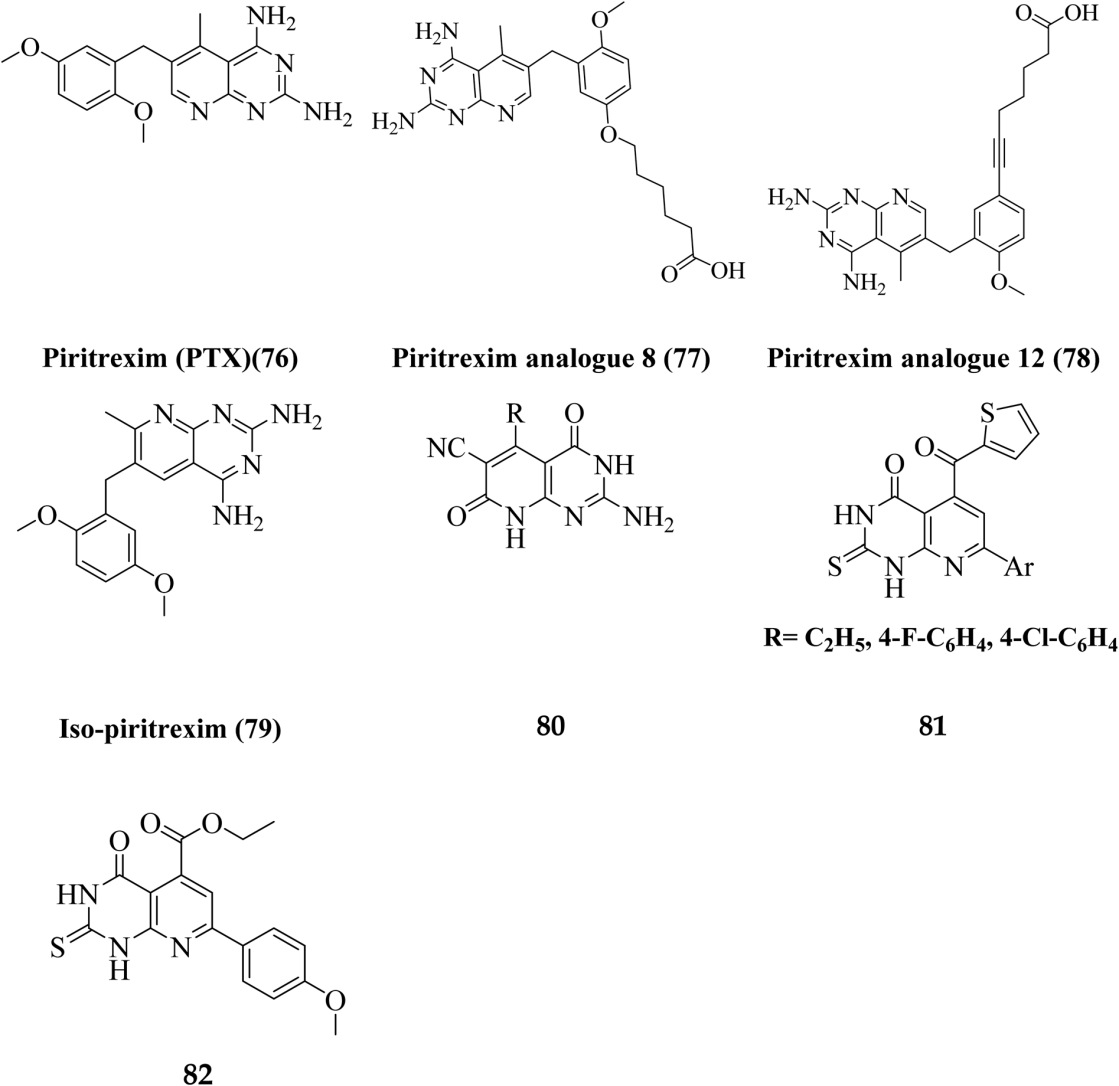
Pyrido[2,3-*d*]pyrimidine derivatives reported as antitumor agents.

Literature review revealed, Piritrexim (76), and its analogues 77–82 (having pyrido[2,3-*d*]pyrimidine pharmacophore) showed excellent antitumor activity (Fig. 17).^27,52–61^

**Fig. 18:**
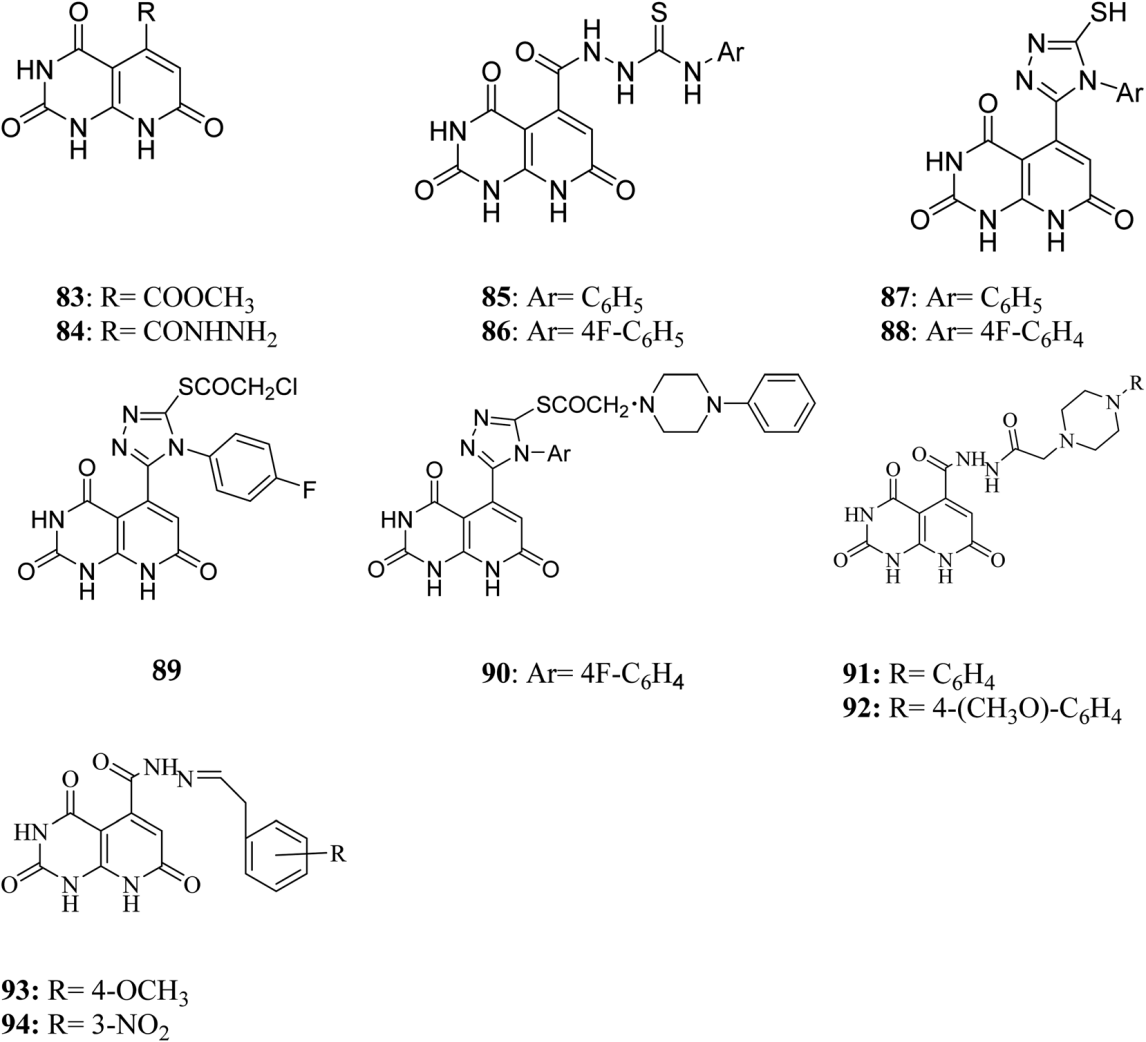
Pyrido[2,3-*d*]pyrimidines reported as antitumor agents.

Gineinah *et al.* designed and synthesized pyrido[2,3-*d*]pyrimidine derivatives; this scaffold was then hybridized with one or more heterocyclic moieties (piperazines or triazoles).^62^ Despite structural alterations to the beginning molecule 83, the examined compounds' principal moiety (pyridopyrimidine) led to the conservation of biological activity. While adding the *N*-phenylthiosemicarbazide moiety, compound 85, at position five of the pyridopyrimidine, increased activity compared to carbohydrazide 84, adding the electron-withdrawing fluoro group to compound 86 at position four of the phenyl ring decreased activity, transferring the ester to acid hydrazide, compound 84, decreased activity. In general, compounds 87 and 88 were found more active than their non-cyclized counterparts, compounds 85 and 86, demonstrating that cyclization of thiosemicarbazide-containing compounds into [1,2,4]-triazoles resulted in more substantial and more robust antitumor activity. Furthermore, compound 89 had lower activity than compounds 87 and 88, indicating that the free sulfanyl group (–SH) had greater potency. In contrast to compound 89, compound 90 was very active as an anticancer drug, implying that the piperazine moiety enhanced antitumor activity. Similarly, including a piperazine moiety in compounds 91 and 92 resulted in high activity (Fig. 18).

In contrast, the 1-phenylpiperazine, a methoxy group at the fourth position, decreased absorbance, rendering molecule 92 more efficient than molecule 91. This demonstrated that enhanced activity may occur when a piperazine moiety is modified at place four with a phenyl ring modified with an electron-donating compound, but not when an electron-withdrawing group or even with an unsubstituted group. It has been demonstrated that compound 93's methoxy group on fourth place of the Schiff base aromatic ring was more active than compound 94's nitro group. The presence of an electron-donating group on an aromatic nucleus is associated with increased anticancer action in part.^62^

### 3.4 BCR-ABL kinase

**Fig. 19:**
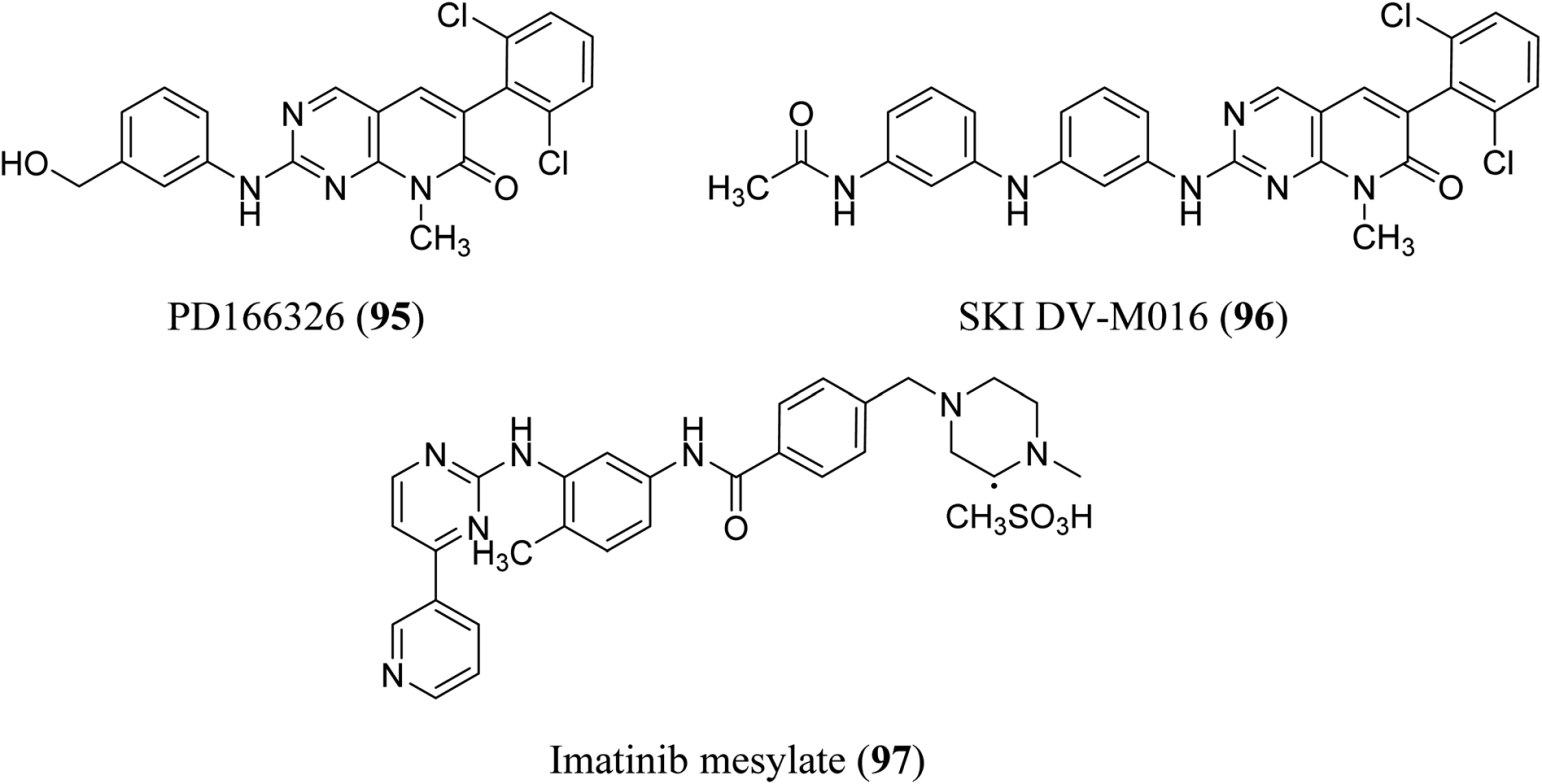
Pyrido[2,3-*d*]pyrimidine derivatives as BCR-ABL kinase inhibitors.

BCR-ABL is a mutation that results from the interaction of two genes, BCR and ABL. It is also known as a fusion gene. The BCR and ABL are present on chromosomes 22 and 9, respectively. When portions of the BCR and ABL genes break off and swap locations, the BCR-ABL mutation occurs. BCR-ABL genes were found in patients with certain types of leukemia and also detected in almost all patients with chronic myeloid leukemia (CML).^63^ Loss of auto-inhibition and constitutional activation of ABL1 may occur when the mutant BCR-ABL1 protein arises, with subsequent effects on signalling pathways related to cell cycle and apoptosis, such as the RAS/RAF/MEK/ERK pathway, the JAK2/STAT pathway, and the PI3K/AKT/mTOR pathway. Finally, it promotes the malignant transformation of hematopoietic cells.^64^ pyrido[2,3-*d*]pyrimidine are effective tyrosine kinase c-Abl and Bcr/Abl inhibitors. At an IC_50_ 2.5 nM, PD180970 reduced Bcr/Abl tyrosine phosphorylation and triggered death in the human CML cell line K562 ^43^. PD180970 was also effective against imatinib mesylate-resistant K562 cells and Ba/F3-P210 cells expressing BCR/ABL kinase domain mutations observed in imatinib mesylate-resistant CML patients.^65^ Compared to imatinib mesylate, PD173955 suppressed the growth of the Bcr/Abl cell line R10(−) with an IC_50_ of 2.5 nM.^66^ PD166326 (95) was the most potent inhibitor of CD34+ CML progenitor development among the six pyridopyrimidines studied, with an IC_50_ around 4-fold lower than PD173955. With an IC_50_ of 300 pM in K562 cells, PD166326 (95) reduced proliferation^66^ and demonstrated action against numerous imatinib mesylate-resistant Bcr/Abl mutants.^66,67^ western blot analysis of Bcr-abl autophosphorylation on K562 cells revealed that PD166326 (95) inhibited Bcr-abl activity in cells. In these cells, IC_50_ for the suppression of Bcr-abl autophosphorylation was 1 nm as compared with 100 nm for STI571. The biological activity and potency of PD166326 (95) was tested in K562 cells in cell growth assays. With an IC_50_ of 0.3 nm, compound 95 suppressed K562 cell growth. Additional hematopoetic and epithelial cell lines were only inhibited at two to three log higher doses with IC_50_s in the 0.8–2 nm range, indicating that PD166326's powerful biological activity was highly selective for Bcr-abl-driven cells.^68^ Bubnoff *et al.* tested 13 different pyridopyrimidine derivatives against wild-type as well as mutant BCR-ABL. All the compounds decreased the Bcr-Abl dependent phenotype more effectively than imatinib and inhibited Bcr-Abl kinase activity (Table 1). PD166326 (95) and SKI DV-M016 (96) were the most active compounds. Surprisingly, these drugs repressed Bcr-Abl H396P, a mutant with an activation loop, just as well as wild-type Bcr-Abl (Fig. 19).^65^

**Table 1:** Bcr-Abl kinase inhibitory activity of compounds 95 and 96

Name	IC_50_ value (nM) (mean ± SE)
SKI DV-M016(96)	4.2 ± 0.6
PD 166326(95)	4.3 ± 0.8

#### 3.4.1 Mechanism of action of BCR-ABL inhibitors:-

**Fig. 20:**
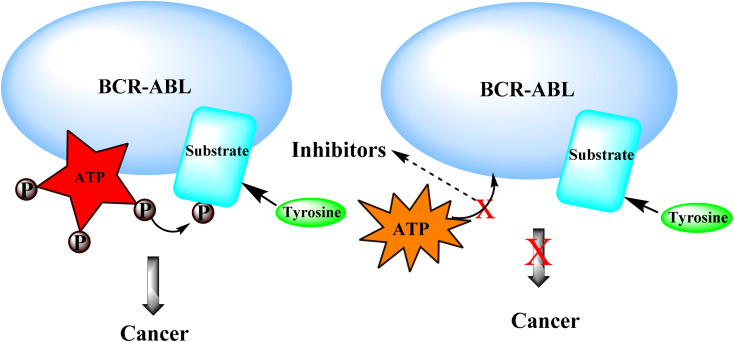
Mechanism of action of BCR-ABL inhibitors.

BCR-ABL inhibitors compete with adenosine triphosphate (ATP) for the same binding site inside the BCR-ABL protein's kinase domain. When ATP is present in the binding site, BCR-ABL can catalyze the phosphorylation of tyrosine residues on substrate proteins. These BCR-ABL inhibitors (Imatinib mesylate) have a structure similar to ATP and can bind to the BCR-ATP-binding ABL's region. Because BCR-ABL inhibitors lack the necessary phosphate groups given by ATP, phosphorylation of tyrosine residues on substrate proteins is impossible. Unphosphorylated tyrosine residues in substrate proteins prevent them from adopting the required conformation for binding to effector molecules. As a result, downstream signal transduction pathways important for CML leukemogenesis are blocked (Fig. 20).^69^

### 3.5 p38 mitogen-activated protein kinases

**Fig. 21:**
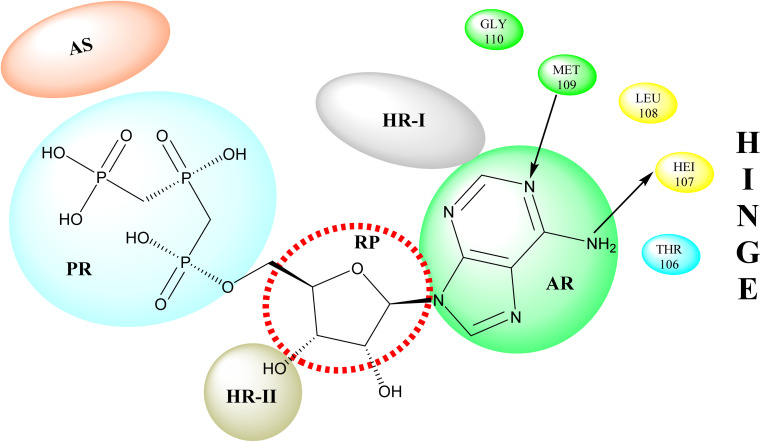
Representation of ATP within p38a MAPK binding site of APT. AS: allosteric site; RP: ribose pocket; AR: adenine region; HR-I and HR-II: hydrophobic regions I and II; PR: phosphate region.

p38 Mitogen-activated protein kinases (p38 MAPKs) are proline-directed serine/threonine kinases that regulate pro-inflammatory signalling networks and are involved in the production of cytokines such as tumour necrosis factor-a (TNF-a) and interleukin-1b (IL-1b).^70,71^ Four isoforms of p38 MAPK have been found, namely α, β, γ, and δ, with p38a MAPK being the earliest identified, best described, and most significant isoform involved in the inflammatory response.^72^ The N- and C-terminal domains of p38a MAPK form the walls of a deep cleft in which the ATP cofactor is allocated in a pocket known as the ATP binding site.^73^ The adenine ring of ATP formed hydrogen bonds with His107 as well as Met109 residues of the p38α MAPK backbone. These two residues are found in the hinge region (residues 106–110), which connects the N and C-lobes, whereas the adenine binding region and ribose pocket, respectively, are located in the adenine binding region and ribose pocket, respectively.^74^ The dual interaction of ATP with the hinge region aligns the two protein lobes to produce a pocket large enough to hold the ATP triphosphate group; this pocket is known as the phosphate binding area and contains highly conserved kinase residues like Glu71 and Asp168.^75^ Three more hydrophobic areas have been discovered adjacent to the ATP binding site but are not directly involved in ATP binding (Fig. 6).^76^ The first section is known as hydrophobic region I (HR-I), and it is defined by Leu75, Lys53, Ala51, Leu104, Ile84, Leu167, and Thr106. It is placed at the back of the ATP binding site (Fig. 21).^77^ Because similar kinases have a bulkier residue at the same position, the tiny size of the gatekeeper residue Thr106 allows access to this pocket, which is essential for kinase inhibitor selectivity. The hydrophobic region II (HR-II), a solvent-exposed hydrophobic area defined mainly through residues Val30, Ile108, Gly110, Ala111, and Asp112, is the second section.^78^ The allosteric site is located next to HR-I and is only accessible when the kinase adopts the catalytically inactive conformation (DFG-out), in which the highly conserved Asp168-Phe169-Gly170 (DFG) motif is flipped “out” in comparison to its active state conformation (DFG-in).^79^

#### 3.5.1 Structure–activity relationship of P38α inhibitors

**Fig. 22:**
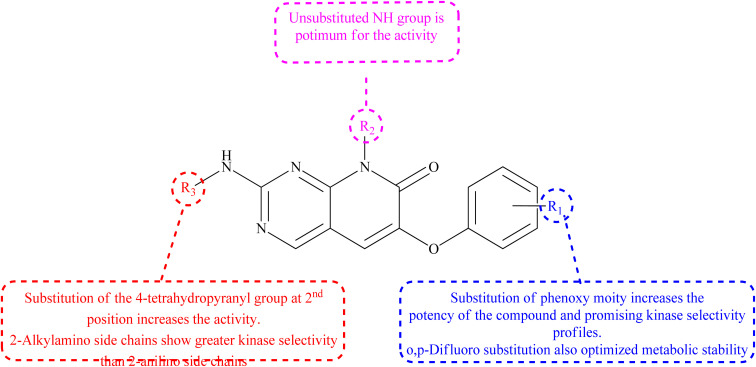
Relationship between structure-modifications and an P38α inhibitors properties.

The 6th-substituent substantially influences inhibitors' pan-kinase selectivity. In terms of potency, this position is also critical. 6-Aryl substituents, such as *o*-chlorophenyl in compound 98, are generally potent compared to 99 (Table 2). Phenoxy substituents were chosen because of their high activities and intriguing profile of kinase selectivity. SAR revealed that the phenoxy group found having tiny ortho and para substituents are preferred over no and meta substitutions. The ortho and para difluoro substitution improved the side chain's metabolic stability. At the 8th-position of the scaffold, many different side chains are allowed, reinforcing the idea that this variable points towards solvent. The kinase selectivity of 2-alkylamino side chains was higher than that of 2-anilino side chains. The molecule's potency was increased by substituting the 4-tetrahydropyranyl group in the second position (Fig. 22).^22^

**Table 2:** IC_50_ (nM) of compounds 98 and 99 as P38α inhibitors

Compound	X	R_1_	R_2_	R_3_	Assay IC_50_ (nM)
					P38α inhibition
98	O	2,4,-diF	CH_3_	4-Tetrahydropyranyl	10 ± 4
99	O	2,4,-diF	CH_3_	CH(CH_2_CH_2_OH)_2_	14 ± 2

**Fig. 23:**
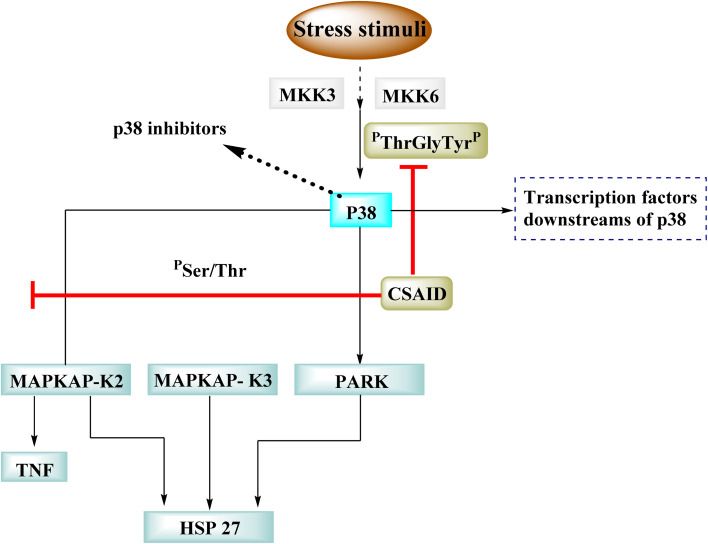
Mechanism of p38 inhibitors.

p38 is one of many kinases implicated in a stress-response signaling pathway similar to but not identical to the MAP kinase cascade. Phosphorylation of threonine 180 and tyrosine 182 by stress signals activates p38. MAPKAP kinase-2 and MAPKAP kinase-3 have been found as downstream targets of p38, which phosphorylates heat shock protein (Fig. 23).^22^

**Fig. 24:**
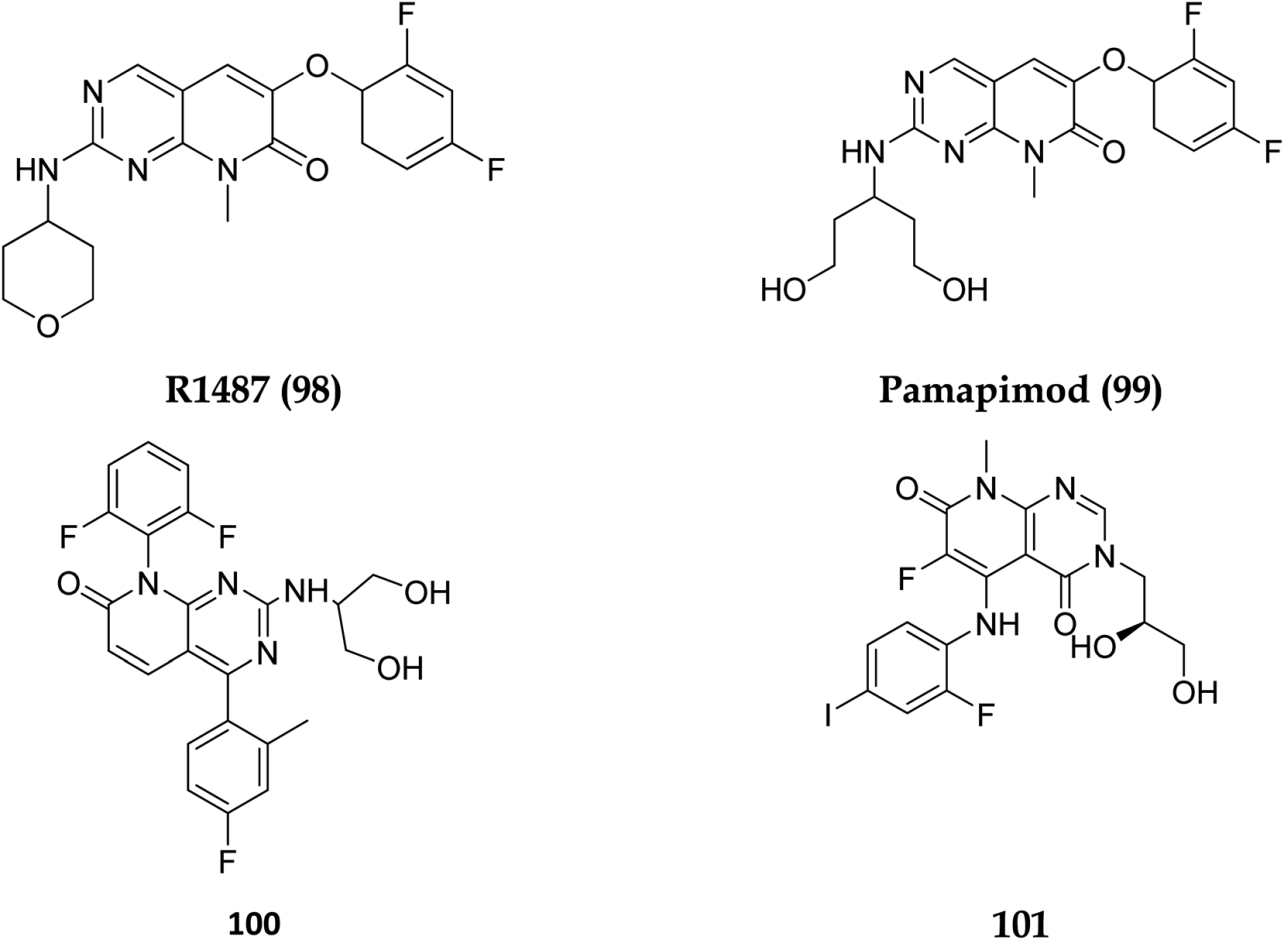
Structures of compounds having p38 inhibitory activity.

Dilmapimod (100, CAS 444606-18-2) is a p38 MAP-kinase inhibitor patented by SmithKline Beecham Corporation (GSK-681323) in 2001 and was used in clinical studies for inflammation, neuropathic pain, and cardiac problems but was eventually ceased due to liver damage.^16^ Clinical research examined TAK-733 (101, CAS 1035555-63-5), a MEK1 and MEK2 (MEK1/2) inhibitor, for advanced non-hematologic malignancies, including metastatic melanoma. It was created and patented by Takeda Pharmaceutical Company in 2007. TAK-733 was tested against non-small cell lung cancer and progressed to Phase I before being recalled off the market in 2015 (Fig. 24).^16^

### 3.6 Phosphatidylinositol 3 kinase and mTOR protein

The phosphatidylinositol (PI) second messengers PI(3)P, PI(3,4)P2, and PI(3,4,5)P3 are produced by phosphoinositide 3-kinase (PI3-KS) enzyme (PIP3). The Class I PI3-KC isoform, which consists of catalytic (p110C) and adapter (p85) subunits is the most common PI3-kinase isoform. By producing 3-phosphorylated phospholipids (PIPs), PI3-KS activates kinases with lipid-binding domains, such as pleckstrin homology (PH) regions, which function as second messengers. For example, Akt as well as phosphoinositide-dependent kinase-1, Akt is moved to the cellular membranes when it links to membrane PIPs, which bind with PDK1 and becomes activated. A tumour suppressor protein, PTEN, dephosphorylates PIP, inhibiting the activation of Akt. The Akt, PDK1, and PI3-Ks are crucial players in controlling numerous cellular functions, including motility, proliferation, survival, and apoptosis. They are also crucial for disease-related molecular pathways, such as cancer, diabetes, and immune-mediated inflammation. Oncogenesis is linked to several components of the PI3K/Akt/PTEN pathway.^80^

**Fig. 25:**
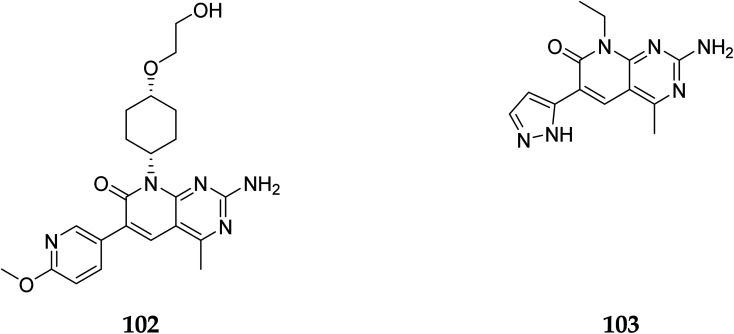
Pyrido[2,3-*d*]pyrimidine derivatives (102 and 103) as PI3K/mTOR inhibitors.

In 2007, Pfizer received a patent for compound 102, inhibiting the mTOR protein and phosphatidylinositol 3 kinases. Due to undesirable effects, it's clinical trial to treat endometrial cancer was stopped in 2012. N68 has also been tested in clinical trials for early (Phase 2) and late breast cancer (Phase 1b).^16^ Finally, Voxtalisib (CAS 934493-76-2) (103), a kinase inhibitor of PI3K and mammalian target of rapamycin in the PI3K/mTOR signaling cascade was patented by Exelis (XL-765) in 2006. In 2018, withdrawal of voxtalisib as a monotherapy and combo therapy for solid tumors (breast and ovarian cancer) took place due to serious side effects (Fig. 25).^16^

In the discovery of anticancer therapies, the mammalian target of rapamycin (mTOR) is a critical target.^81^ The growth factor/mitogenic activation of the phosphatidylinositol 3-kinase (PI3K/Akt) signalling pathway activates this key regulator of cell growth and proliferation, frequently dysregulate route responsible for diseases. Serine and theonine kinases are the 2 mTOR found in the mammalian body. It's about 289 kDa in size and belongs to the evolutionary family. The eukaryotic PI3K like kinase (PIKK) family of proteins is highly conserved.^82–84^

#### 3.6.1 Mammalian target of rapamycin pathway

**Fig. 26:**
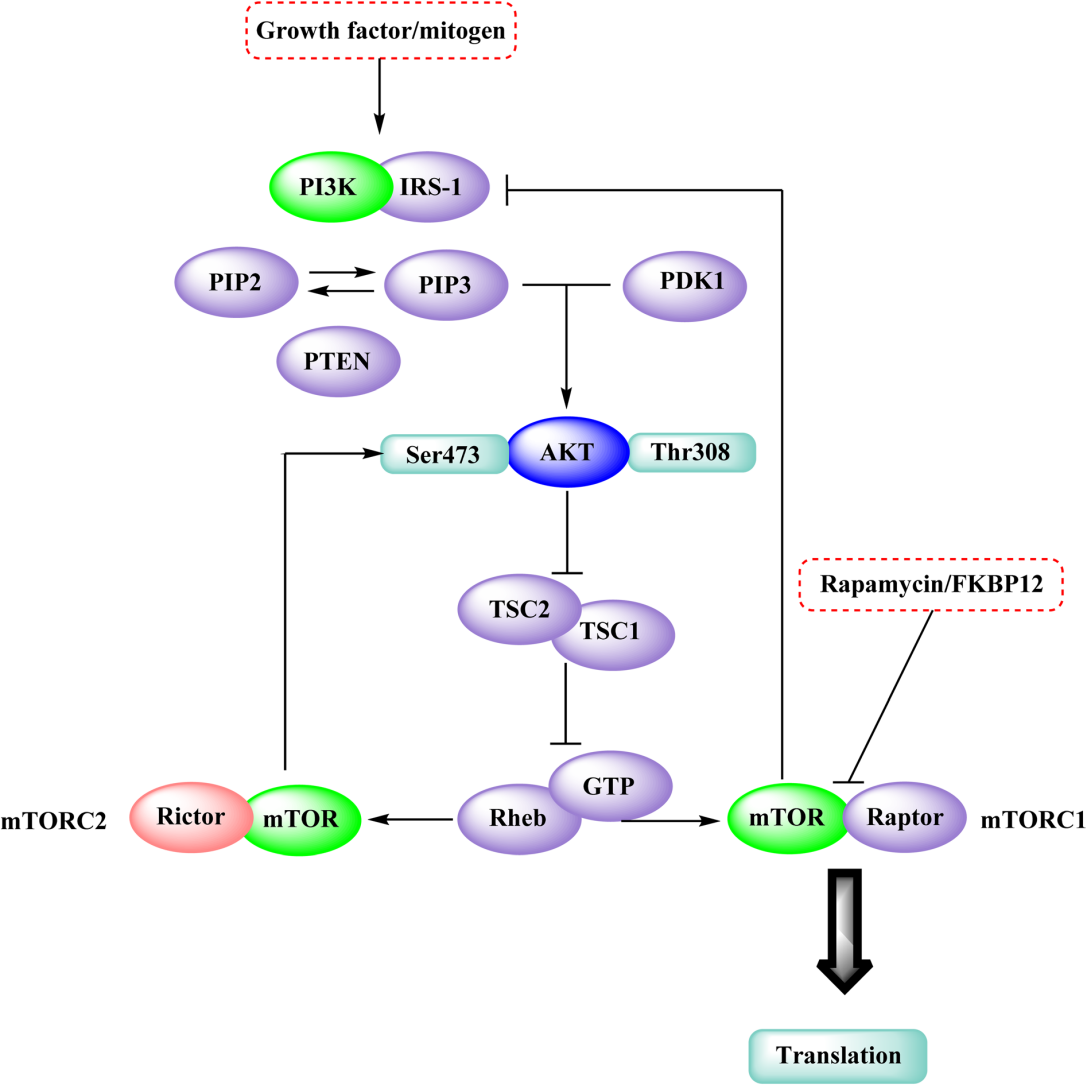
The mTOR kinase inhibition in the mTOR-PI3K-AKT pathway.

The rapamycin and its analogues (RAD001, CCI-779, AP23573) bind to the FKBP12/rapamycin complex binding domain (FRB) and suppress signaling downstream substrates p70S6K and 4E-BP1.^84–86^ LY294002 and wortmannin, potent but non-specific PI3K inhibitors, have also been demonstrated to block the kinase activity of mTOR by targeting the protein's catalytic domain.^87^ According to the Brunn *et al.* and Edinger *et al.* mTOR is divided into two complexes: mTORC1, a rapamycin-sensitive complex that signals to p70S6K and 4E-BP1, and mTORC2, a rapamycin-insensitive complex that signals to Akt.^87,88^ Therefore, direct targeting of the mTOR kinase domain would suppress signalling *via* both mTORC1 and mTORC2 and such a drug would have a distinct pharmacological spectrum than rapamycin (Fig. 26).^84^

#### 3.6.2 Structure–activity relationship of mTOR inhibitors

**Fig. 27:**
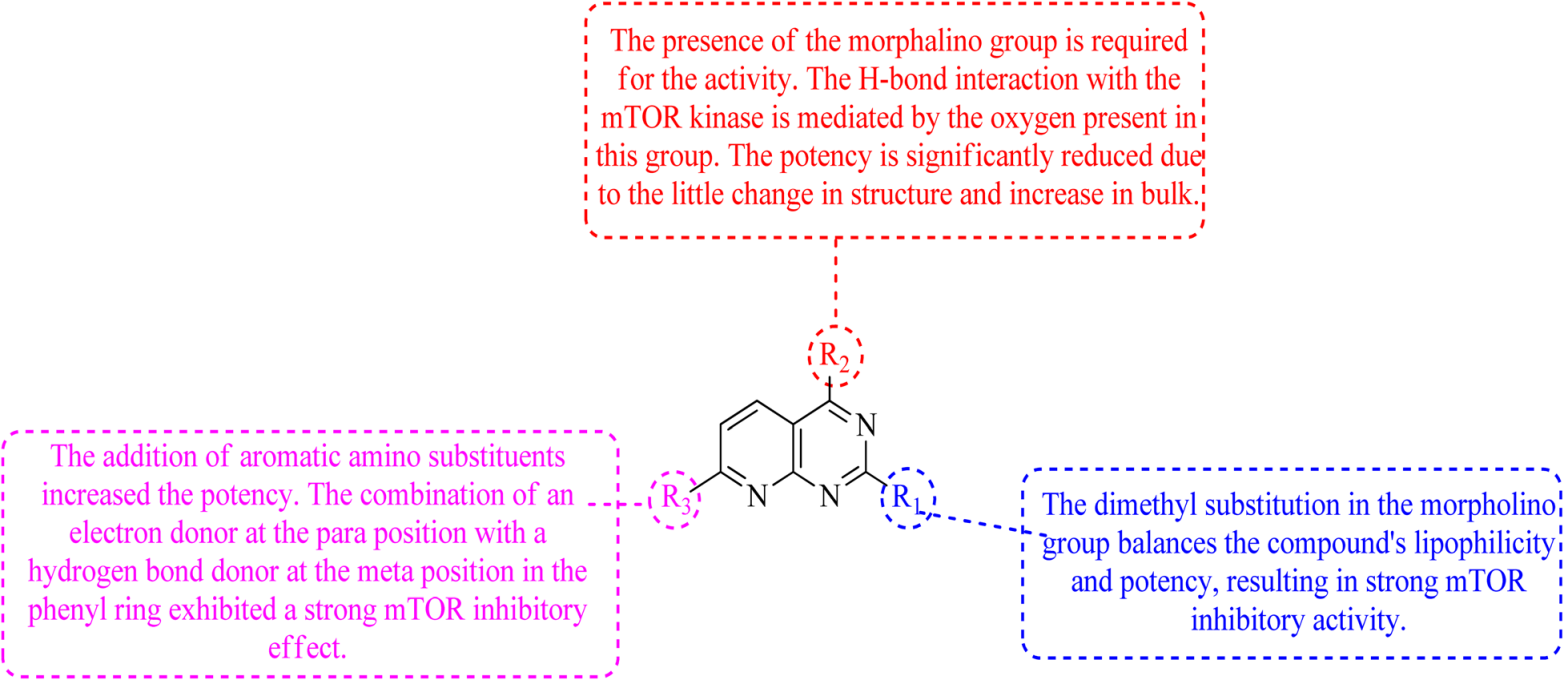
SAR of mTOR inhibitors.

The dimethyl substitution at C_2_ of the morpholino group balances the compound's, resulting in strong mTOR inhibitory activity (Table 3). The presence of the morpholino group is required for mTOR inhibitory activity. The oxygen in this group mediates the H-bond interaction with the mTOR kinase. The potency is significantly reduced due to the slight change in structure and increase in bulk. The combination of an electron donor at the para position with a hydrogen bond donor at the meta position in the phenyl ring exhibited a strong mTOR inhibitory effect (Fig. 27).^84^

**Table 3:** Pyrido[2,3-*d*]pyrimidine derivatives with mTOR inhibitory activity

S. No.	Compound	R_1_	R_2_	R_3_	mTOR inhibition IC_50_ (μM)
1	104	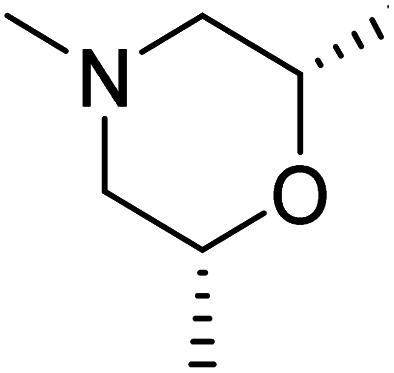	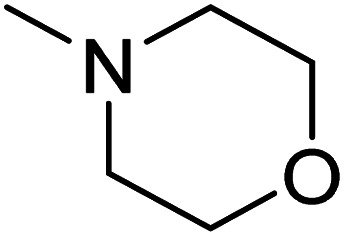	—	1.3
2	105	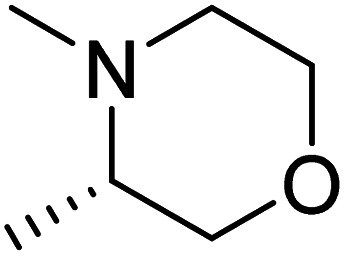	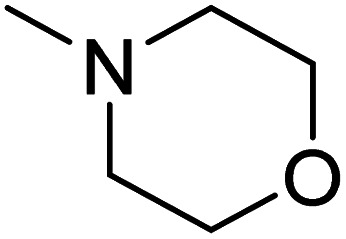	—	0.69
3	106	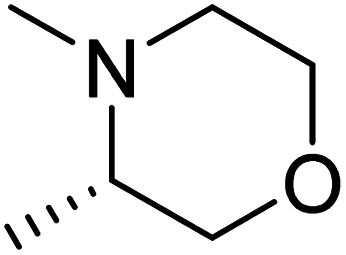	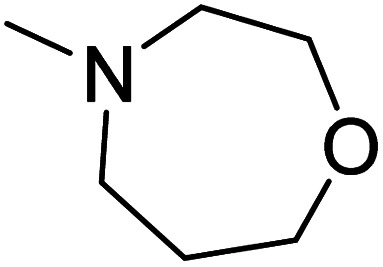	—	4.8
4	107	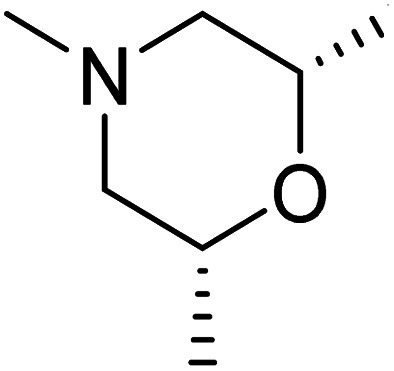	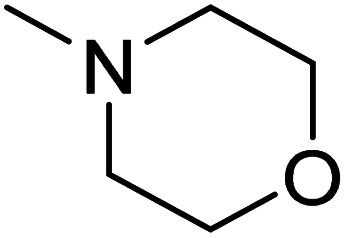	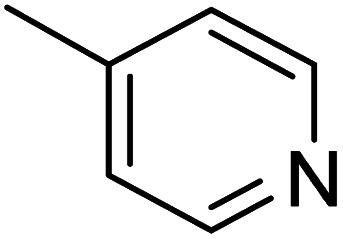	0.22
5	108	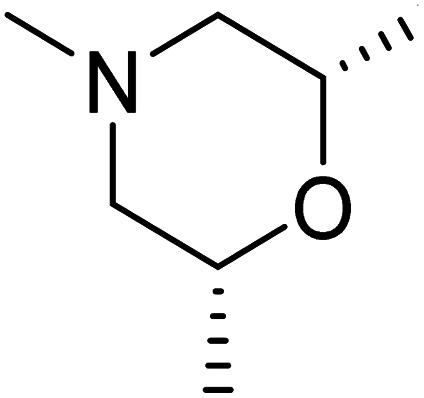	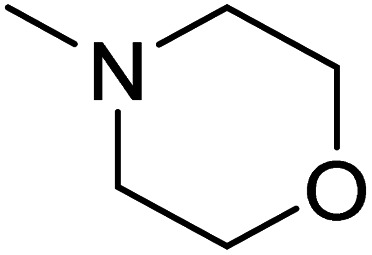	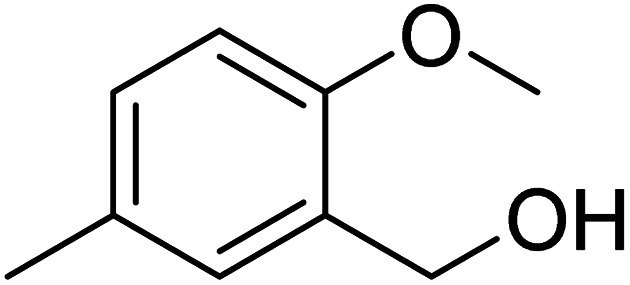	0.016

The KU-63794 (compound 108) indicated that mTOR selectivity has been achieved compared to other PIKK family members. Furthermore, it exhibited no significant activity when tested on 200 kinases (non-PI3K related) at a concentration of 10 μM. U87MG glioblastoma cells were treated with the KU-63794 (108) for 2 h to see whether it inhibited both mTORC1 and mTORC2 complexes in the cellular environment. Using a western blot, the phosphorylation of the direct mTORC2 substrate Akt (Ser473) and the indirectly mTORC1 downstream signalling S6 ribosomal protein (Ser235/236) was determined. The IC_50_s were 0.10 and 0.15 μM for each of the endpoints.

### 3.7 Targeted inhibition of dihydrofolate reductase

**Fig. 28:**
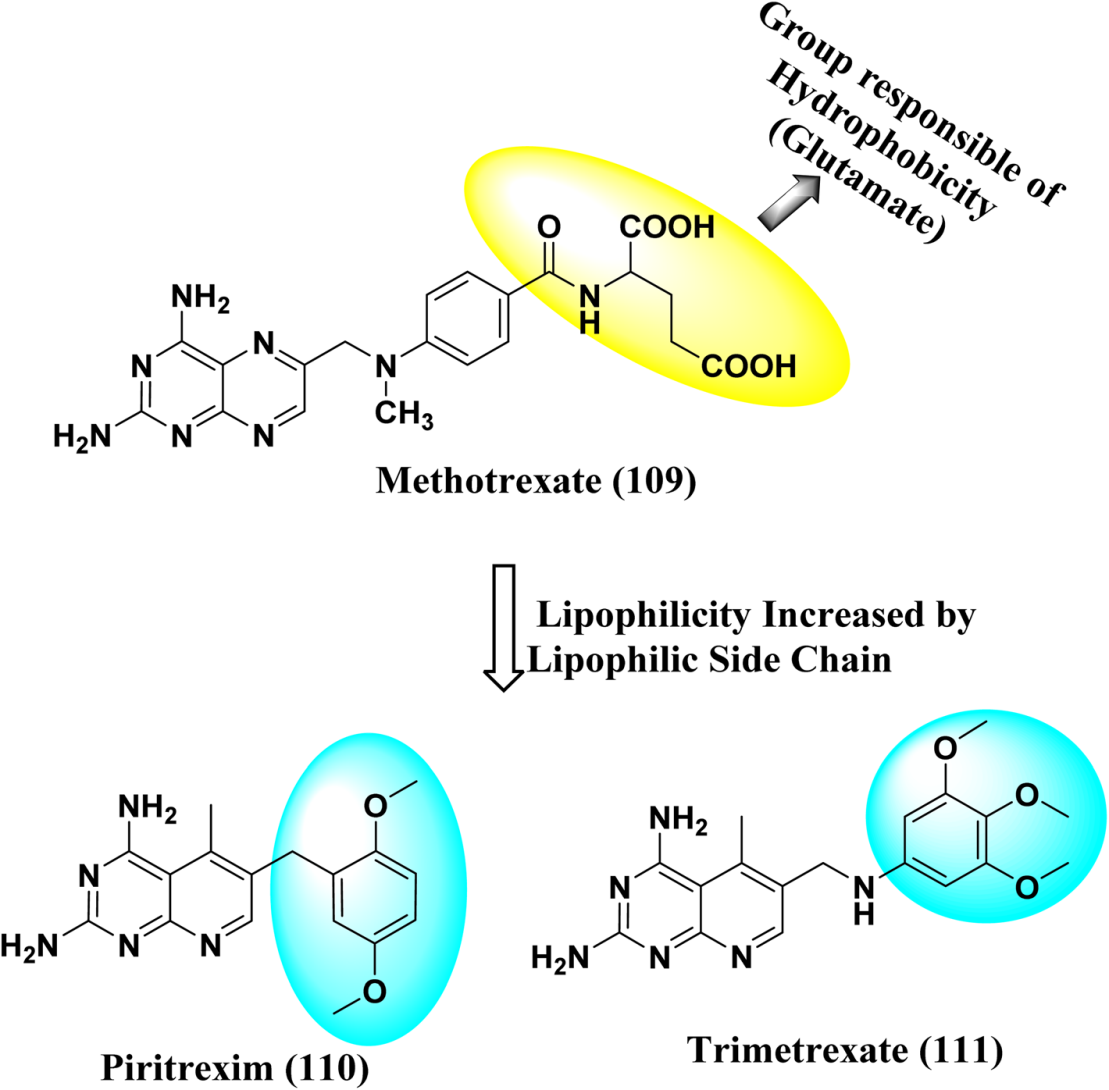
Methotrexate (109) and pyrido[2,3-*d*]pyrimidine derivatives (110 and 111) DHFR inhibitors.

NADPH (Nicotinamide adenine dinucleotide phosphate) serves as a cofactor for dihydrofolate reductase (DHFR), which transforms dihydrofolate (DHF) into tetrahydrofolate (THF). THF is necessary to form *de novo* purine and thymidylate (TMP) during cell growth.^89^ Inhibiting DHFR activity causes cell death,^90^ making DHFR an essential target enzyme in developing chemotherapy.^91^ Antifolates are categorized into two types: traditional/Classical: Due to the inhibitor's hydrophilic characteristic and glutamate moiety (which prevents the inhibitors from diffusing into cells), an active transport mechanism is needed. However, in the non-classical form, substituting the hydrophilic glutamate moiety with lipophilic groups improves its passive diffusion into cells.^92,93^ Dihydrofolate reductase (DHFR), specific kinases, such as the tyrosine-protein kinase transforming protein Abl or MAP kinases, and the biotin carboxylase are the most commonly described biological targets of pyrido[2,3-*d*]pyrimidine derivatives (Fig. 28).^18^

**Fig. 29:**
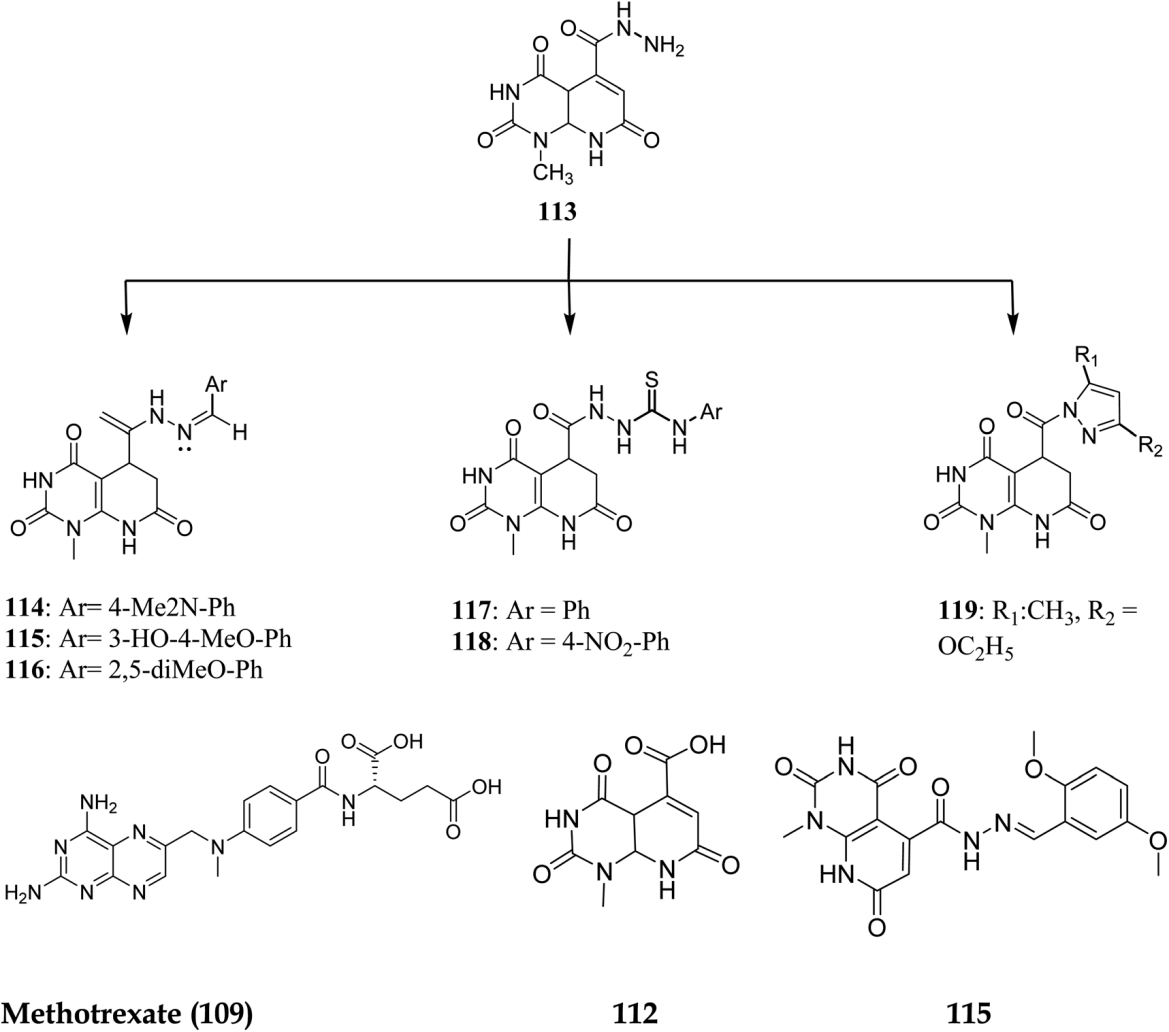
Pyrido[2,3-*d*]pyrimidine derivatives as DHFR inhibitors.

Walaa M. *et al.* (2019) synthesised pyrido[2,3-*d*]pyrimidine derivatives and evaluated their *in vitro* antitumor activity against five human cells lines: HePG2, MCF-7, PC3, HCT-116, and HeLa, using doxorubicin as a positive control. Compounds 112–119 (Fig. 29) showed the highest antitumor effects and were tested for enzymatic inhibition of the dihydrofolate reductase [compared to the reference medication methotrexate (MTX)] to explain the likely mechanism of action of the observed anticancer activity.^94^ Molecule 115 had the highest inhibitory activity at IC_50_ of 6.5 μM among all the tested molecules (IC_50_ of MTX = 5.57 μM, Table 4).

**Table 4:** *In vitro* and *in silico* activity of compound 115 against DHFR

Compound no.	Kinase IC_50_ (μM)	Docking score (kcal/mol)	*In vitro* IC_50_ (μM)
			MCF-7
115	6.5	−14.2	5.66 ± 0.4
MTX(109)	5.57	−14.7	

#### 3.7.1 Structure–activity relationship study of DHFR inhibitors

**Fig. 30:**
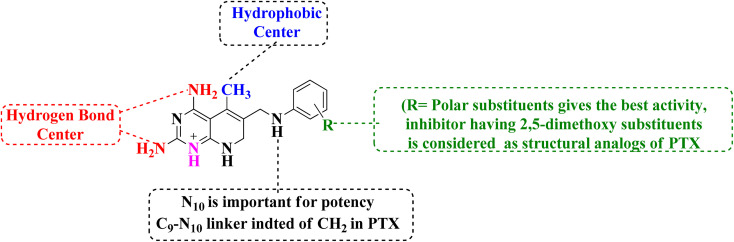
SAR of DHFR inhibitors.

SAR study indicated that pyrido[2,3-*d*]pyrimidine scaffold was essential for activity. If the R was replaced with 2,5-dimethoxy substituents, it acted as PTX, and modification at NH position enhanced the activity. Both NH_2_ groups were responsible for hydrogen bonding (Fig. 30).

### 3.8 Apoptosis inducer

#### 3.8.1 Cyclin-dependent kinase (CDK)

In cancer, the cell-cycle regulatory mechanism, which is necessary for cellular division, is commonly disturbed, resulting in tumour growth. CDK4/6 inhibition inhibits cell proliferation and suppresses DNA replication in malignancies with functioning retinoblastoma. Many positive and negative regulators closely control cell division and the cell cycle. Mitogenic cues activate and upregulate cyclin D, getting complexed with CDK4/6, caused phospholylation leads to retinoblastoma, allowing bound E2F transcription factors to be released for the cell to divide. The cell cycle is positively regulated by CDKs and their cyclin partners, whereas the cell cycle is negatively regulated by retinoblastoma, other tumour suppressors (p16INK4b, p18INK4c, and p19INK4d), and the CDK-interacting protein/kinase inhibitory protein (Cip/Kip) family. Palbociclib is a CDK4/6 inhibitor effective in preclinical breast cancer models.^95^ After identifying cyclin-dependent kinases as essential regulators of cell proliferation, Pfizer Inc. developed palbociclib. Warner–Lambert received a patent for palbociclib in 2003. Palbociclib (pyrido[2,3-*d*]pyrimidin-7(8*H*)-one) interferes with the cell cycle. This inhibitor of CD4 kinase was picked out of the pyridopyrimidine class due to its intriguing structural and pharmacological characteristics. The FDA authorized it to treat HR-positive, advanced HER2-negative, or metastatic breast cancer in March 2015.^95^ Protein kinases, CDKs (cyclin-dependent kinases), are crucial for cellular processes. They participate in vital physiological processes like transcription and the cell cycle. Based on sequence similarities, the human genome contains 21 genes that encode CDKs, and five genes that encode a more distant group of proteins have been identified as CDK like (CDKL) kinases. The current CDK nomenclature includes 11 classical CDKs (CDK1-11), two newly proposed family members (CDK12-13), and additional proteins whose names are based on the presence of a cyclin-binding element (PFTAIRE and PCTAIRE proteins) or simply on sequence similarity to the original CDKs, such as CDC2-like kinases (CDC2L) or Cell cycle-related kinases (CCRK).^96^

Apoptosis means allowing cells to die in a controlled manner. Apoptosis is a tightly controlled process in which cells self-destruct and eliminate undesired or diseased cells. The cell genome fractures, shrinks, and the cell portion disintegrate into smaller apoptotic entities during apoptosis.^97^ Cancer cells can potentially resist apoptosis, resulting in uncontrolled cancer development.^98^ The standard cellular mechanism called apoptosis keeps the ratio of healthy living cells to healthy dying cells in equilibrium. As a result, triggering apoptosis in cancer cells may be viewed as a cancer therapy strategy that does not harm healthy cells and prevents tumour resistance. As a result, finding novel drugs that target any phase of the cell cycle that leads to apoptosis might be a promising anticancer strategy.^99^

#### 3.8.2 Cyclin-dependent kinase (CDK4/6)

**Fig. 31:**
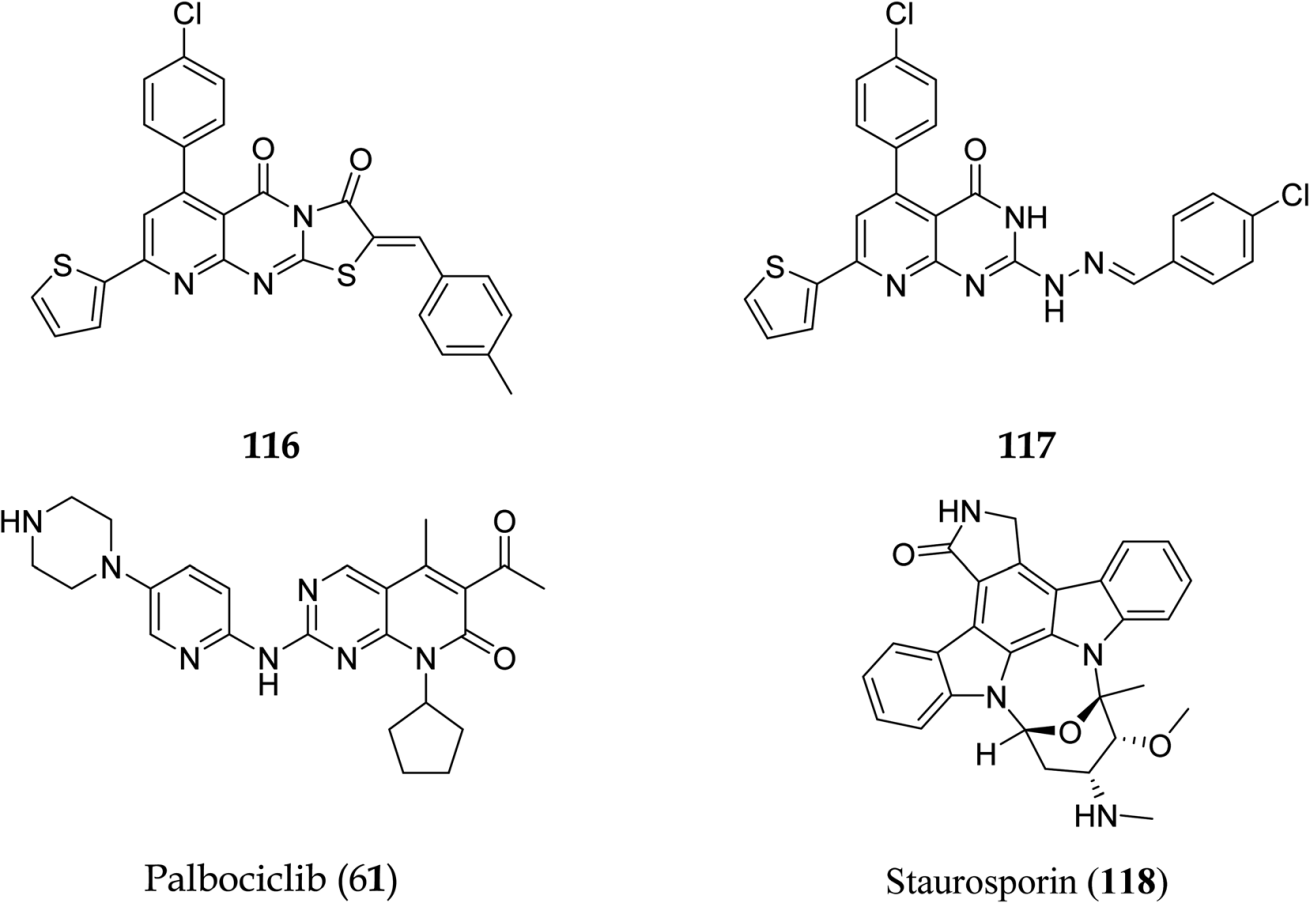
Pyrido[2,3-*d*]pyrimidine derivatives with CDK 4/6 inhibitory activity.

Pyrido[2,3-*d*]pyrimidine is a critical component in various bioactive compounds, including anticancer medicines, attributed to cyclin-dependent kinase inhibition.^100,101^ Palbociclib is a CDK4/6 inhibitor licensed by the US Food and Drug Administration to treat breast cancer.^102^ Safinaz *et al.*, in 2019, prepared pyrido[2,3-*d*]pyrimidines derivatives and evaluated their cytotoxicity activity against various cell lines, including MCF-7, PC-3, and luA-549 and compared the results with doxorubicin. They synthesized different pyrido pyrimidine derivatives and checked their *in vitro* activity against human cancer lines (breast MCF-7, lungs A-549, and prostate PC-3),^103^ Cell cycle analysis,^104^ Apoptosis assay,^105^ Caspase activation assay,^106^ RT-PCR for (Bax, Bcl2,p53, and CDK4/6), and compound 116 showed excellent activity against Caspase-3 (Table 5). Furthermore, compounds 116 and 117 showed an excellent docking score and primary amino acid interaction with selected protein (Table 6 and Fig. 31). Compounds 116 and 117 were observed to promote apoptosis in PC-3 and MCF-7 cells *via* activating Bax, p53, and caspase-3, down-regulating Bcl2 and inhibiting CDK4/6. Furthermore, compound 116 directly inhibited CDK6 with an IC_50_ of 115.38 nM, whereas compound 117 inhibited it with an IC_50_ of 726.25 nM (Table 7).^41^

**Table 5:** Effect of compound 116 on Caspase-3

Compound no	Caspase-3 conc. (Pg ml^−1^)	Optical density
Control	43.45	0.103
116	603.4	0.741

**Table 6:** Docking scores of compounds 116, 117, and 118 with PDB ID 5L21

Compound no	Docking score (kcal mol^−1^)	Interactions with amino acids
Palbociclib (61)	−12.02	Asp163, Val101
116	−13.24	Val77, Phe164, Val101, Phe98, Asp163
117	−15.40	—

**Table 7:** Activity of compounds 116 and 117 on cyclin-dependant kinase-6

Compound no	IC_50_ (nM)
116	115.38
117	726.25
Staurosporin (118)	92.78

#### 3.8.3 CDK2 roles in the cell cycle and cancer development

CDKs are key regulators of the cell cycle,^107^ and the proper regulation of CDK activity is crucial for the ordered execution of the cell cycle.^108^ CDK2/cyclin E expression abnormalities have been seen in colorectal, ovarian, breast, and prostate cancers.^109^ CDK2 is involved in many stages of the cell cycle, including DNA repair, gene transcription, the G1-S transition, and G2 progression modulation.^110–112^

#### 3.8.4 The function of CDK2 in the proliferation of cells: -

**Fig. 32:**
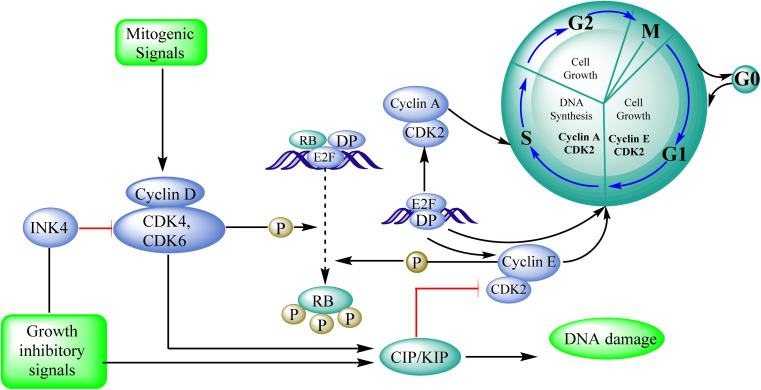
Role of CDK2 in the cell cycle progression.

CDK2 is involved in cell cycle control, transcription activity, and tumor epigenetic alterations. Certain malignancies, such as breast cancer,^113^ colo-rectal cancer,^114^ glioblastoma,^115^ and melanoma^116^ may benefit from CDK2 inhibition (Fig. 32).

**Fig. 33:**
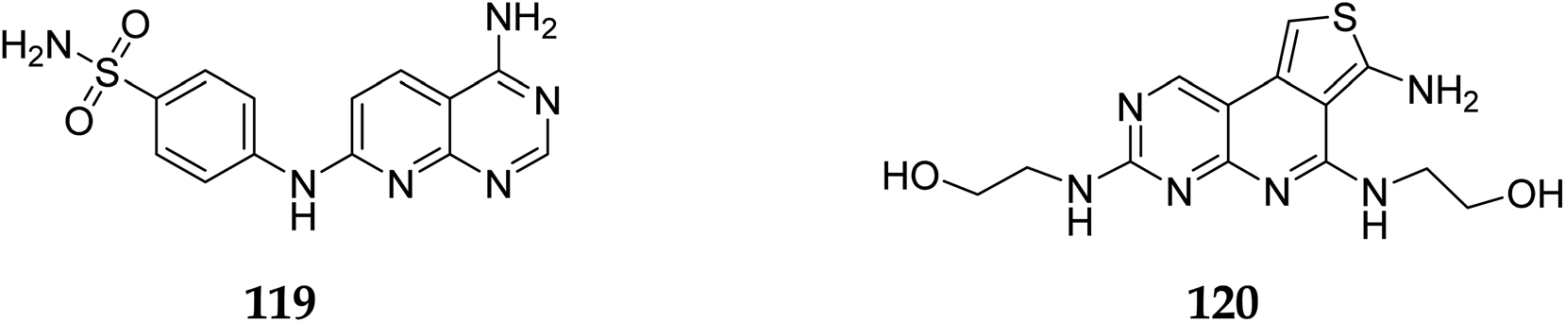
Pyrido[2,3-*d*]pyrimidine derivatives as CDK2 inhibitors.

Cyclin-dependent kinase promotes the progression of the G1 phase into the S phase by phosphorylation of the retinoblastoma protein (RB). The E2F family of transcription factors is activated by the hyperphosphorylation of the RB, which reduces its capacity to regulate growth. Growth repressive signals promote CDK inhibitors from the INK4 and CIP/KIP families, which block G1-S progression. Cyclin and dependent kinase complexes are activated by mitogenic signals.^117,118^ Diaa *et al.*, 2011 designed and synthesized 2,5,7-trisubstituted pyrido[2,3-*d*]pyrimidines by introducing a variety of substituents at C-2, C-5, and C-7 of the pyridopyridine ring for a potent and selective inhibitors of CDK2 (competitive inhibitors of ATP binding site) and tested them for CDK2, EGFR inhibitory activities, and cell growth inhibitory activities.^10^ Using the design of structural scaffolds addressing ATP interactions, they created pyrido[2,3-*d*]pyrimidine scaffolds (CDK2 inhibitors) using the X-ray crystalline structure of CDK2 (PDB ID: 1HCK). The CDK2 inhibitory and anti-proliferative activity is due to the side chains at the 2nd and 7th positions. The CDK2, CDK4, EGFR, and anti-proliferative activities of the Pyrido[2,3-*d*]pyrimidine derivatives were assessed against SNU638, A431, and HCT116, and the results showed that compounds 119 and 120 were more active than roscovitine with IC_50_ values of 0.3 and 0.09 μM, respectively, which shows that compound 120 has the good anti-proliferative activity. The compounds did not show any significant activity against the EGFR, which shows their selectivity against the CDK2 (Fig. 33).^10^

#### 3.8.5 Structure activity relationship of CDK2 inhibitors

SAR studies relealed that 7-amino group's lipophilic function (LF), such as the benzene, and sulfonamide group, significantly increased the CDK2 inhibitory effect. Further, small (LF) with an electron donating group on position 2 of the pyrido[2,3-*d*]pyrimidine moiety was required for CDK2 inhibitory activity.

### 3.9 Phosphodiesterase inhibitors

Phosphodiesterase 3 is also known as the cyclic GMP-inhibited PDE family. PDE3A and PDE3B are the two genes in the family, each has numerous splice variants. Harrison *et al.*, Macphee *et al.*, Maurice and Haslam *et al.* were the first to discover PDE3A as a significant PDE isoform in bovine heart and platelets.^119^ PDE3 enzymes play a role in modulation of cardiac and vascular smooth muscle contractility. PDE3 inhibitors were once examined for the treatment of heart failure. However, they are no longer being studied for that due to undesired arrhythmic side effects. The PDE3 inhibitor milrinone is licensed for intravenous usage in the treatment of heart failure.^120^ Abadi *et al.*, 2013 synthesized 7-(3-bromophenyl)-5-(2-ethoxyphenyl) pyrido[2,3-*d*]- pyrimidin-4-amine and 7-(3-bromophenyl)-5-(2-methoxyphenyl)pyrido[2,3-*d*]- pyrimidin-4(3H)-one by cyclization of 2-amino-6-(3-bromophenyl)-4-(2-ethoxyphenyl) nicotineonitrile and 2-amino-6-(3-bromophenyl)-4-(2-methoxyphenyl)- nicotinonitrile by refluxing with formamide and formic acid respectively.^121^ PDEs could be promising targets for tumour cell growth control and apoptosis induction. These are; (I) modification of cyclic nucleotide signaling is known to contribute to one of the pathways regulating growth and operation of tumour cells. (II) Various PDE isozymes are discovered in various cancer tissues, (III) non-selective PDE inhibitors like theophylline or aminophylline affect the proliferation of cancer cell lines.^122^ HMG (Human malignant melanoma) has been found to have PDE3 activity, indicating that it might be a potential target for antineoplastic medications. However, the PDE3-specific inhibitors trequinsin and cilostamide did not affect HMG cell growth.^123^ The expansion of colon cancer cells was associated with the upregulation of PDE3B mRNA as well as protein in HT-29 cells. Through the dose-dependent activation of PDE3B, cyclic phosphatidic acid (cPA) reduced Akt phosphorylation, lowering HT-29 cell proliferation. As a result, the cPA-PDE3B-cAMP pathway plays an essential role in colon cancer growth, and cPA could be exploited for colon cancer targeted therapy.^124^

#### 3.9.1 Mechanism of action of phosphodiesterase inhibitors

The cyclic adenosine monophosphate (cAMP) and cyclic guanosine monophosphate (cGMP) signalling systems are among the earliest signalling systems discovered, and are involved in a variety of physiological processes such as visual transduction, cell proliferation and differentiation, cell-cycle regulation, gene expression, inflammation, apoptosis, and metabolic function.^125^ Adenylyl-cyclase and guanylyl-cyclase produce cyclic nucleotides when G-protein-coupled receptors and molecules like natriuretic peptide or nitric oxide have been activated. cAMP then activates cAMP-dependent kinase (PKA) and other proteins, regulating biological processes like cell differentiation and proliferation. Proteins are phosphorylated when cGMP activates the cGMP dependent protein kinase (PKG), which is involved in physiological mechanisms like ion channel conductance as well as cell death. Phosphodiesterases 3 (PDEs) regulate cAMP and cGMP intracellular concentrations and their diverse biological consequences by catalyzing their hydrolysis.^122^

**Fig. 34:**
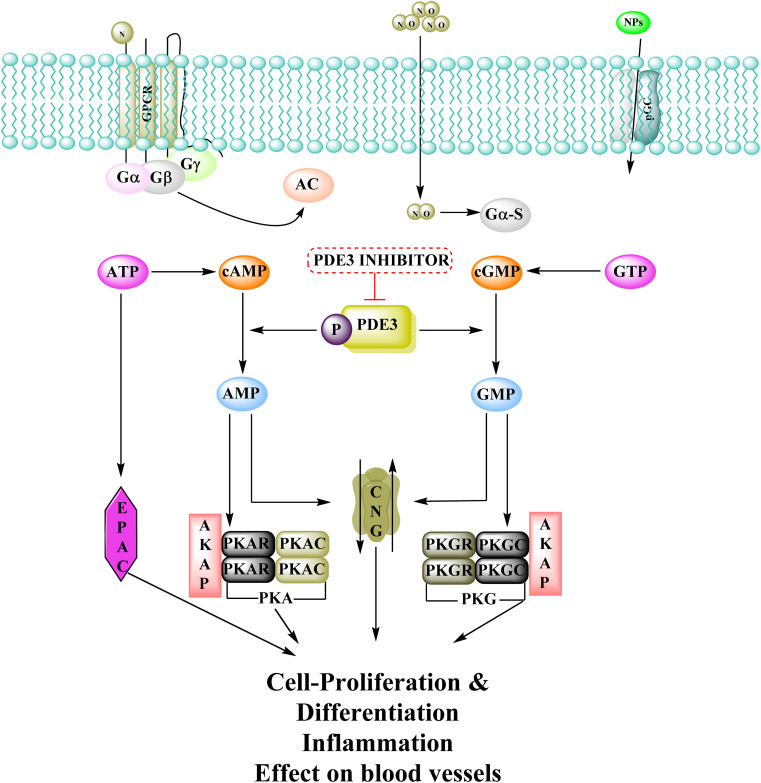
Phosphodiesterase 3 signalling pathway and its inhibitors. NO: nitric oxide; PKA: protein kinase A; GPCR: G-protein-coupled receptors; NPs: atrial natriuretic peptide and B-type natriuretic peptide; AC: adenylate cyclase; EPAC: exchange protein activated by cAMP; PKG: protein kinase G; CNG: cyclic-nucleotide-gated ion channels; pGC: particulate guanylyl cyclase; sGC: soluble guanylyl cyclase.

PDEs can be expressed by every cell type, and their location is crucial in controlling cAMP or cGMP cellular functions.^126^ Interference with the cAMP/Cgmp signalling pathway has been linked to tumour growth. Increased intracellular cAMP/cGMP may suppress tumour growth and hence be a protective mechanism against tumour progression.^3^ PDE inhibitors that limit cAMP or cGMP hydrolysis have thus been identified as possible anticancer medicines (Fig. 34).^125^ However, some cancers, such as adenocarcinoma, can stimulate cell proliferation when cAMP levels are elevated.^127,128^

**Fig. 35:**
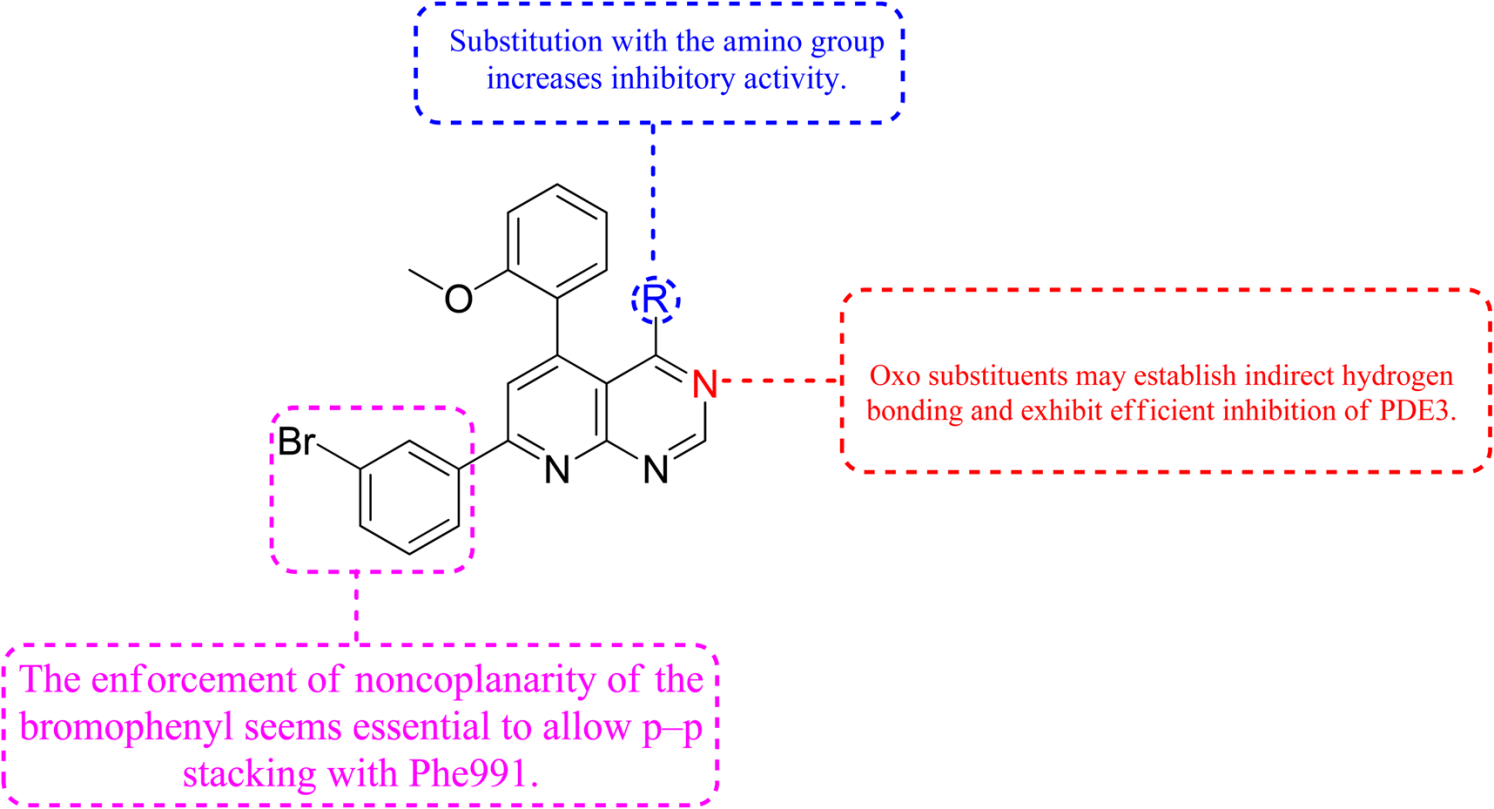
SAR of phosphodiesterase inhibitors.

A cyclized pyridopyrimidone analog (122) has an IC_50_ as low as 1.34 μM. With cAMP and cGMP as substrates, these two (121, and 122) molecules efficiently inhibited PDE3. This indicates that for the best anticancer action, suppression of both cAMP, as well as cGMP hydrolysis to boost intracellular concentrations of both signalling molecules may be necessary (Fig. 35). Utilizing cGMP as the substrate, compound 121 had an IC_50_ of 10.9 μM and suppressed PDE3 (Table 8). Creating substrate-specific inhibitors for dual-functioning enzymes like PDE3 is a novel development that opens the way to modify the expression of one substrate instead of another, which may have implications for drug discovery in terms of safety and efficacy. An adaption of the IMAP (Fluorescence polarization phosphodiesterase assay) was used to detect PDE activity.

**Table 8:** Inhibitory activity of compounds 121 and 122 on Cell Line (HT-29) and phosphodiesterase 3B

Compound no.	HT-29		PDE3 inhibition			
	% Growth inhibition at 50 μm	IC_50_ μm	% PDE3 inhibition at 50 μm		IC_50_ μm	
			cAMP	cGMP	Camp	cGMP
121	86.10	18.27	48	85	ND	10.91
122	97.98	1.34	62	78.6	24.21	10.75
Milrinone (123)	10	>50	77	95	11.4	3.6

#### 3.9.2 Relationship between structure-propeties and anticancer activities

**Fig. 36:**
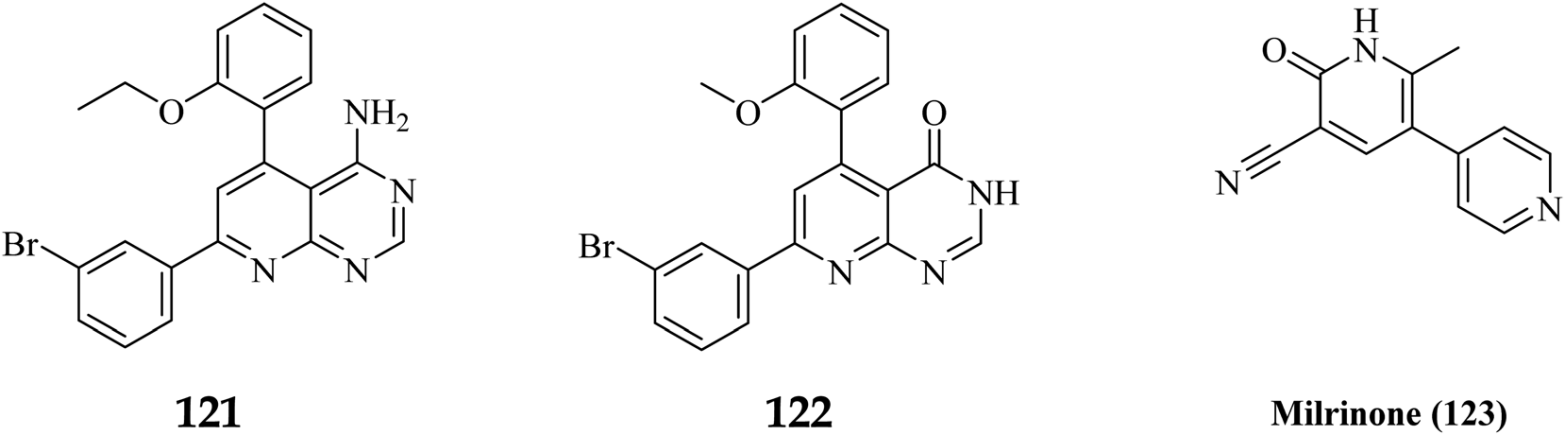
Pyrido[2,3-*d*]pyrimidine derivatives, 122 and 123 with milrinone as phosphodiesterase inhibitors.

The cyclized pyridopyrimidone derivative 121 reduced the tumour cell proliferation, with an IC_50_ of 1.34 μm (Fig. 36). With cAMP and cGMP as substrates, these three drugs efficiently inhibited PDE3. This indicated that the best strategy to treat cancer may be to limit both cAMP as well as cGMP hydrolysis to increase intracellular concentrations of both secondary messengers. The positive control milrinone's inability to cause apoptosis in the HT-29 cell line despite its dual inhibition of PDE3 and growth suggested that the other PDE and off proteins may contribute to the anticancer activity. Alkoxy and Bromo substituents at the ortho positions of the phenyl at positions 4 and 6, respectively, are believed to be responsible for the non-coplanarity between the two aryls and pyridone. The enforcement of bromophenyl non-coplanarity appears to be necessary for stacking with Phe991. Replacement of the oxygen atom of cyano-2-pyridones with an amino group may create the foundation for substrate-selective pharmacological regulation of this significant class of therapeutic targets (Fig. 35).^121^

### 3.10 Gene Kirsten rat sarcoma viral oncogene homolog inhibitors

The Gene Kirsten rat sarcoma viral oncogene homolog is known as KRAS. It belongs to the group of epidermal growth factor receptor (EGFR) kinase. This multi-component signaling system helps to regulate cell growth, division, survival, and death by relaying signals from the outside to the inside of the cell. An essential signal for cellular differentiation in normal cells is the interaction of the epidermal growth factor to its target, mainly on the cell membrane. There are two more signals in the process including tyrosine kinase enzymes as well as a protein encoded by the KRAS gene, instead of separately promoting cell proliferation, the route's components typically cooperate to control cell division and growth. KRAS mutations are frequently linked to resistance to targeted medicines and poor outcomes in cancer patients, yet after more than three decades of research, no selective KRAS inhibitor has been authorized. However, in some tumors, EGFR becomes active even when EGF is not present, resulting in uncontrolled cell proliferation and division. EGFR or tyrosine kinase enzyme inhibitors are frequently used to treat these malignancies. However, an alteration in the KRAS gene found in a few of these malignancies leads to an abnormal K-Ras enzyme. The uncontrolled protein is constantly active and can promote cell growth despite the lack of signals from EGFR as well as other tyrosine kinase enzymes. Inhibitors of EGFR or tyrosine kinases will be ineffective in such tumours.^129–131^ KRas is the most often mutated oncogene among all types of human cancer. After the initial discovery of Ras oncogenes in 1982, In Ras-driven cancer, no approved medication specifically targets Ras.^132^ Ras proteins are chemical messengers that alternate between an active GTP-bound as well as an inactive GDP-bound state to control a number of cellular functions. Guanine nucleotide exchange factors (GEFs) enhance activation, and GTPase-activating proteins (GAPs) inactivates Ras protein by catalyzing GTP hydrolysis and are involved in these state changes.^133,134^ Ras protein mutations result in anomalies in the switch mechanism, a significant contributor to tumorigenesis. RAS mutations are present in three of the most lethal kinds of human malignancy, or around 25% of human tumors, including pancreatic cancer, and colon and lung cancer. KRas is the most frequently altered isoform of the Ras proteins (85%), followed by NRas (11%) and HRas (4%), with the most common mutations occurring at amino acid positions G12, G13, and Q61.^132^ Mutations at positions 12 and 13, which cause a steric clash with the catalytic arginine finger donated by the GAPs, are thought to be oncogenic. In contrast, mutations at position 61 disrupt the coordination of a nucleophilic water molecule, resulting in an accumulation of active GTP-bound Ras proteins in cells.^135,136^ The Shokat laboratory discovered the switch-II pocket in 2013, creating an opportunity for the creation of the KRasG^12C^ inhibitors, which is investigated in the trials. A library of 480 tethering compounds was screened for covalent binding to KRasG^12C^ using a disulfide-fragment-based screening method.^137^

**Fig. 37:**
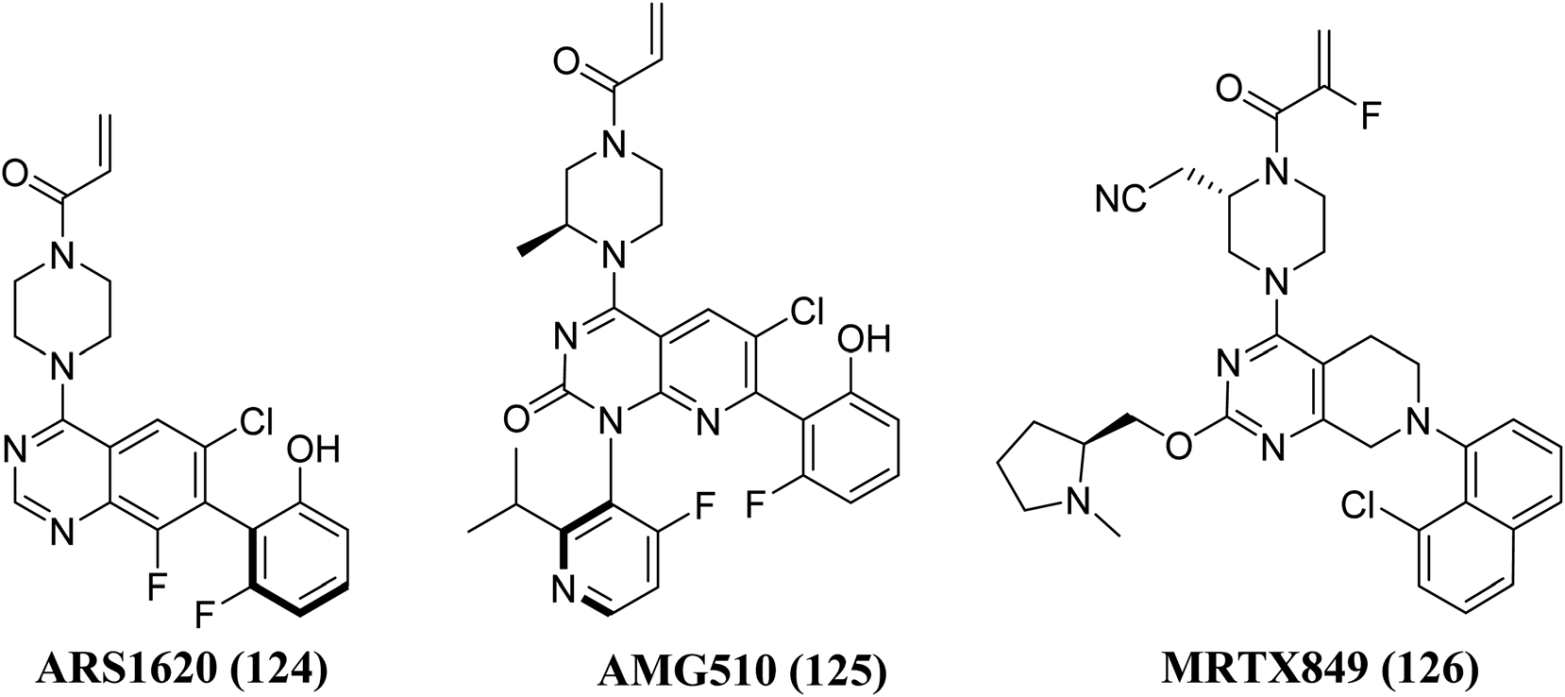
Chemical structure of pyrido[2,3-*d*]pyrimidine derivatives 125, with 124, & MRTX849 (126) as KRAS inhibitors.

Structure–activity-relationship (SAR) investigations of 6H05, compound 6Ostrem, and KRAS^G12C^ in the GDP state resulted in a co-crystal structure with KRAS^G12C^. Because crystallographic study revealed that the ligand does not attach to the nucleotide-binding pocket (S-IIP), it was given the switch-II pocket and placed near the activator binding switch-II. S-IIP is positioned between the core-sheet and switch-II, and when ligand binding occurs, it undergoes significant conformational changes to form a separate pocket, whereas switch-I conformation remains intact from the GDP-bound state.^132^ Many novel covalent allosteric KRAS^G12C^ inhibitors have been created as a result of this pioneering work, including ARS1620 (124),^138^ AMG510 (125),^139,140^ and MRTX849 (126),^141,142^ with AMG510 being the first KRAS^G12C^ inhibitor to reach clinical trials in August 2018 (Fig. 37). The findings of a phase I trial using this medication were recently released, revealing partial responses in 50% of non-small cell lung cancer (NSCLC) patients and stable disease in the majority of colorectal or appendix cancer patients, all of whom carried the KRAS^G12C^ mutation.^143^

**Fig. 38:**
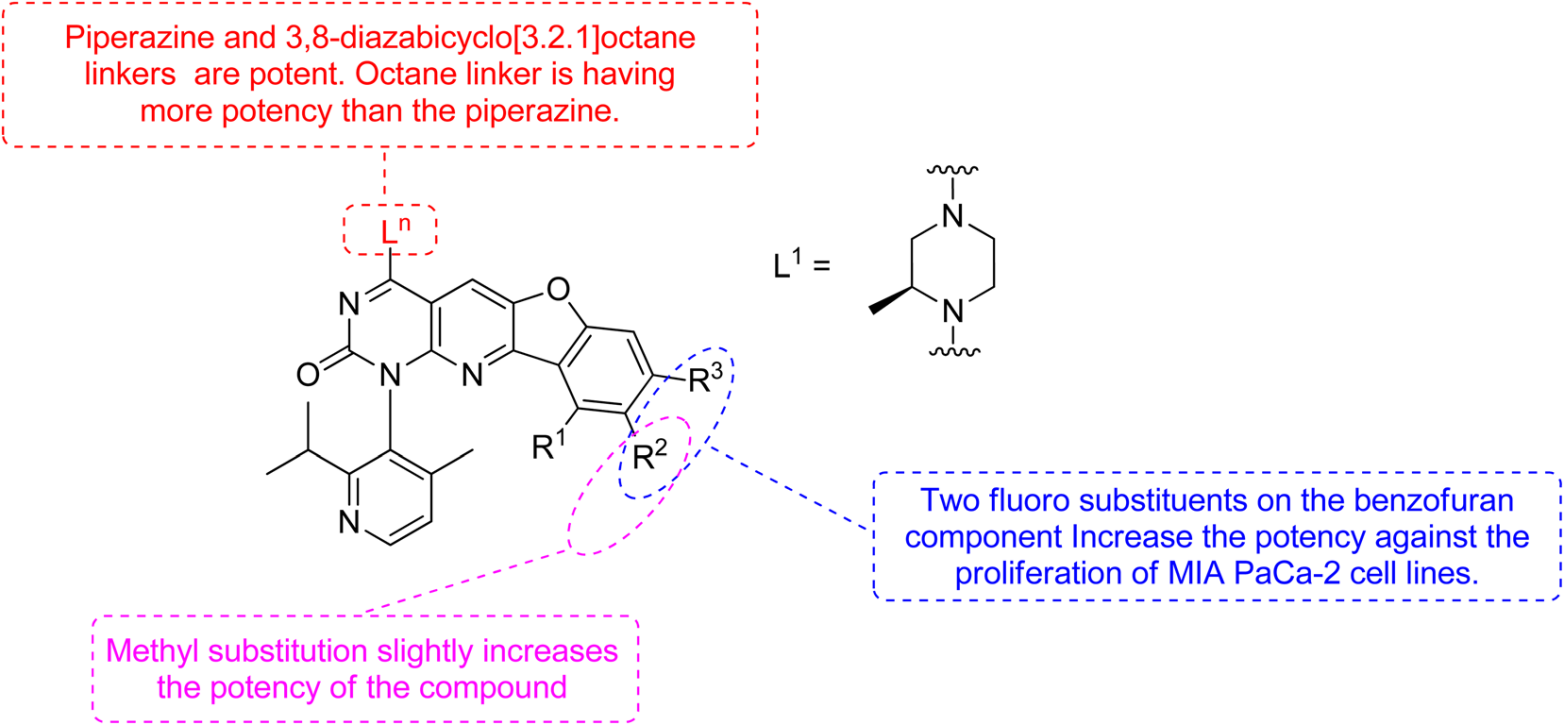
SAR of KRAS inhibitors.

Xiao *et al.*, 2021, designed new KRAS^G12C^ inhibitors using a novel approach. Two sets of pyrido[2,3-*d*]pyrimidine- and pyridinyl N-atom-shifted bicyclic pyrido[4,3-*d*]pyrimidine-containing derivatives of the clinical KRAS^G12C^ inhibitor AMG510 (125) were produced (Fig. 38). MIA PaCa-2 cells, clinical pancreatic cancerous cells with the KRAS^G12C^ mutations, were used to test the tetracyclic derivatives; investigating potential off-target effects, the inhibitory activity on A549 cells carrying other KRAS mutant G12S has also been investigated. Although compound 128 demonstrated considerable efficacy, including an IC_50_ value of 7.97 μM in MIA PaCa-2 cell line expressing KRAS^G12C^, the tetracyclic molecules still exhibited selectivity toward the G12C mutation of KRAS (Table 9). According to the docking study, the shadow connection between 128 in the front entry and an open hydrophobic pocket may be the main reason for its less efficacy. The piperazine derivative was less effective than the 3,8-bicyclo[3.2.1]octane linker in the parent skeleton. However, it was found that the piperizinyl linker, which joins the bond formation warhead towards the tetracyclic nucleus, had some biological efficacy. Compounds 127 and 128 have the most effective R_2_-substituents, such as methyl or fluoro group.^143^

**Table 9:** Antiproliferative effects of 127 and 128 against MIA PaCa-2 cell lines

Compound no	Ln	R_1_	R_2_	R_3_	IC_50_
127	L1	H	CH_3_	H	>10 μM
128	L1	H	F	F	7.97 μM
AMG510 (125)	—	—	—	—	0.029 μM

The more robust character of (pyrido[4,3-*d*]pyrimidin) in comparison to 128 (pyrido[2,3-*d*]pyrimidin), explained by fluorophenol group engaging a hydrophobic pocket and creating an H-bonds to Arg68. Additionally, the fluorophenol molecule appeared to occupy the space by the tiny fluoro group at the C-8 place (as in ARS1620). Only molecule 128 showed minor activity in the MIA PaCa-2 human cell expressing KRAS^G12C^, with IC_50_ of 7.97 μM, whereas the tetracyclic molecules still showed specificity for the G12C form of KRAS. According to structural docking study, the shadow contact of 127 at the front entry and an unoccupied hydrophobic pocket may be the primary cause of its weak potency. In biochemical and cellular experiments, bicyclic compound 128 was found to be substantial inhibitor.^143^

### 3.11 Fibroblast growth factor receptors inhibitors

Several endothelial and tumour cells contain tyrosine kinases called FGFR (fibroblast growth factor receptors). They participate in tumour angiogenesis, tumour cell migration, survival, and proliferation. Many human malignancies have been connected to FGFR overexpression or aberrant activity control. As a result, reducing tumor cell proliferation, survival, and migration, as well as tumor angiogenesis by targeting FGFRs is an appealing technique for developing cancer therapeutic alternatives.^144^ The activation of downstream transduction pathways occurs when the intracellular kinase domain of the FGFRs undergoes transautophosphorylation due to the binding of FGFs, which also causes the dimerization of FGFRs.^145,146^ FGFRs contribute to crucial physiological activities, including cell migration, proliferation, differentiation, and survival, *via* activating downstream signalling pathways.^147–149^

#### 3.11.1 FGFR signaling pathways

**Fig. 39:**
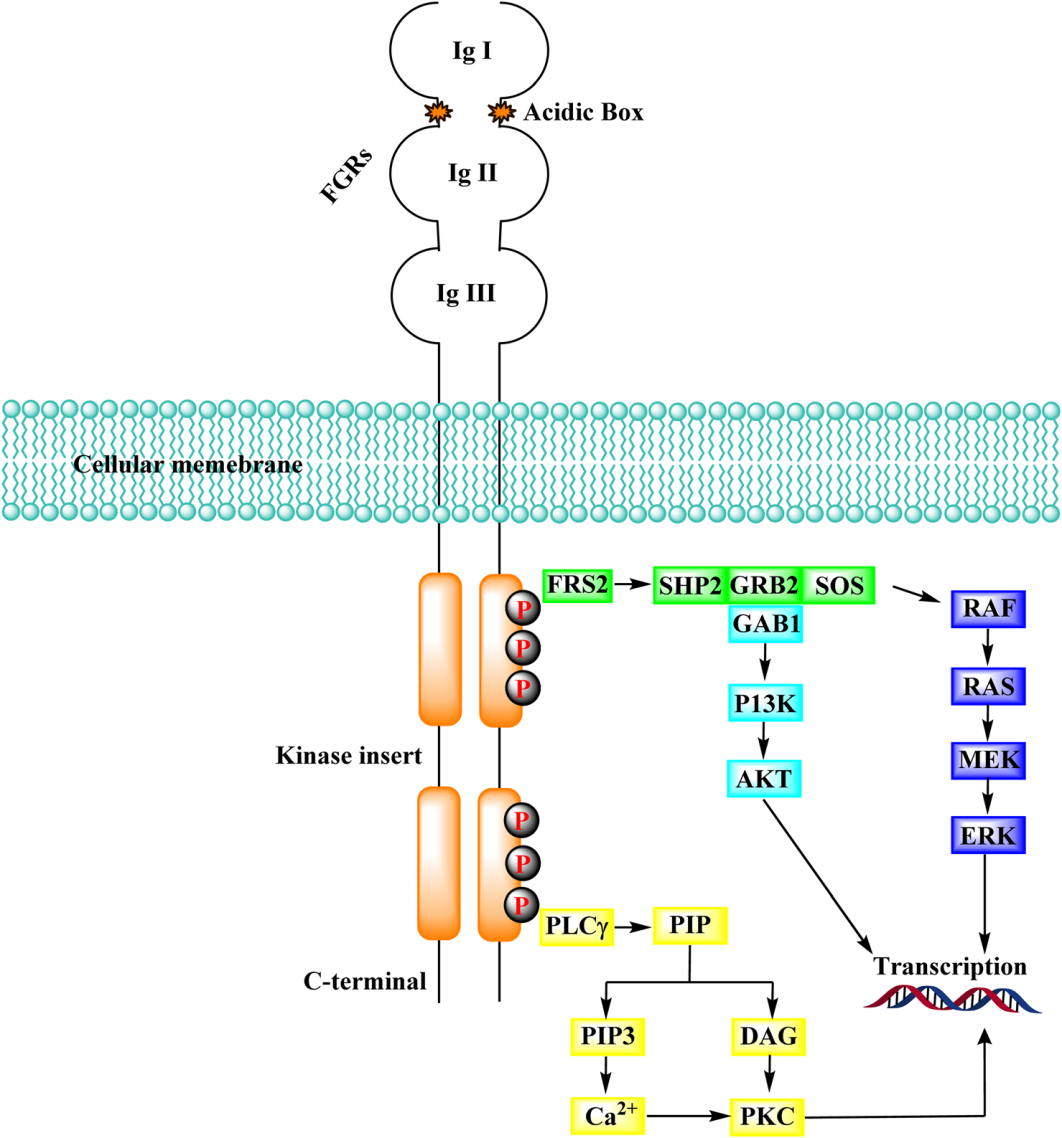
FGFR signaling cascade.

FRS2, a prominent FGFR substrate, binds to the juxta membrane area of FGFR constitutively *via* its phospho-tyrosine binding domain (PTB), regardless of the kinase domain's activity or phosphorylation state. Several tyrosine residues in FRS2 are phosphorylated after FGFR activation and serve as docking sites for the following molecules.^150^ The RAS-MAPK-ERK signalling cascade is repeatedly activated by FRS2. RSA was chacterised by docking with different proteins, including SHP2 (SH2-containing tyrosine kinase phosphatase), GRB2 (growth factor receptor-bound protein 2), SOS (Son of Sevenless). The Src symmetry domain (SH2 domain) in SHP2 as well as GRB2 could bind directly phosphorylated tyrosine residues in GRB2 and FRS2. As a result, either directly or through the creation of the SHP2-GRB2-SOS complex, the GRB2-SOS complex is recruited to FRS2.^151,152^ In the RAS-MAPK-ERK signalling pathway, the complex then initiates a phosphorylation cascade. ERK1/2 is translocated from the cytoplasm to the nucleus after activation. It regulates the activity of various transcription factors to influence cell proliferation, differentiation, and signal transduction, making it one of the most persuasive signalling molecules in this pathway.^153^ When GRB2-related binding protein 1 (GAB1) binds to GRB2's SH3 domain, it can phosphorylate tyrosine itself and be dragged into the complex. Similarly, PI3K with an SH2 domain binds to the phosphorylated tyrosine residues of GRB2, activating the PI3K-AKT signalling cascade. AKT's downstream effector molecules include the well-known mTOR, which is involved in cell metabolism, transcription, and other processes (Fig. 39).^154^

In addition to FGFR, PIP2 (phosphatidylinositol 4,5-bisphosphate) is hydrolyzed by phospholipase C, which connects to a phosphorylated tyrosine in the kinase domain's C-terminus to produce the secondary mediators IP3 (inositol triphosphate) and DAG (diacyl glycerol). When IP3 binds to its receptor on the endoplasmic reticulum, Ca^2+^ is released from intracellular reserves, increasing Ca^2+^ concentration.^155^ DAG activates the PKC signalling pathway when it is coordinated with Ca^2+^, which produces crosstalk with the RAS-MAPK pathway (due to the rivalry between GRB2 and PLCγ) for FGFR binding.^156^

Connolly *et al.* from the Parke-Davis laboratory were the first to identify drugs tailored to target FGFR selectively. The authors used high-throughput screening approaches to find FGFR inhibitors. This led to the discovery of pyrido[2,3-*d*]pyrimidine (129), which inhibited FGFR potently (IC_50_ = 0.45 μM) in a biochemical assay; this indicates that the compound 129 binds at the ATP-binding site, and the molecule behaved in an ATP-competitive fashion.^157^

#### 3.11.2 Structure–activity relationship of FGFR inhibitors

**Fig. 40:**
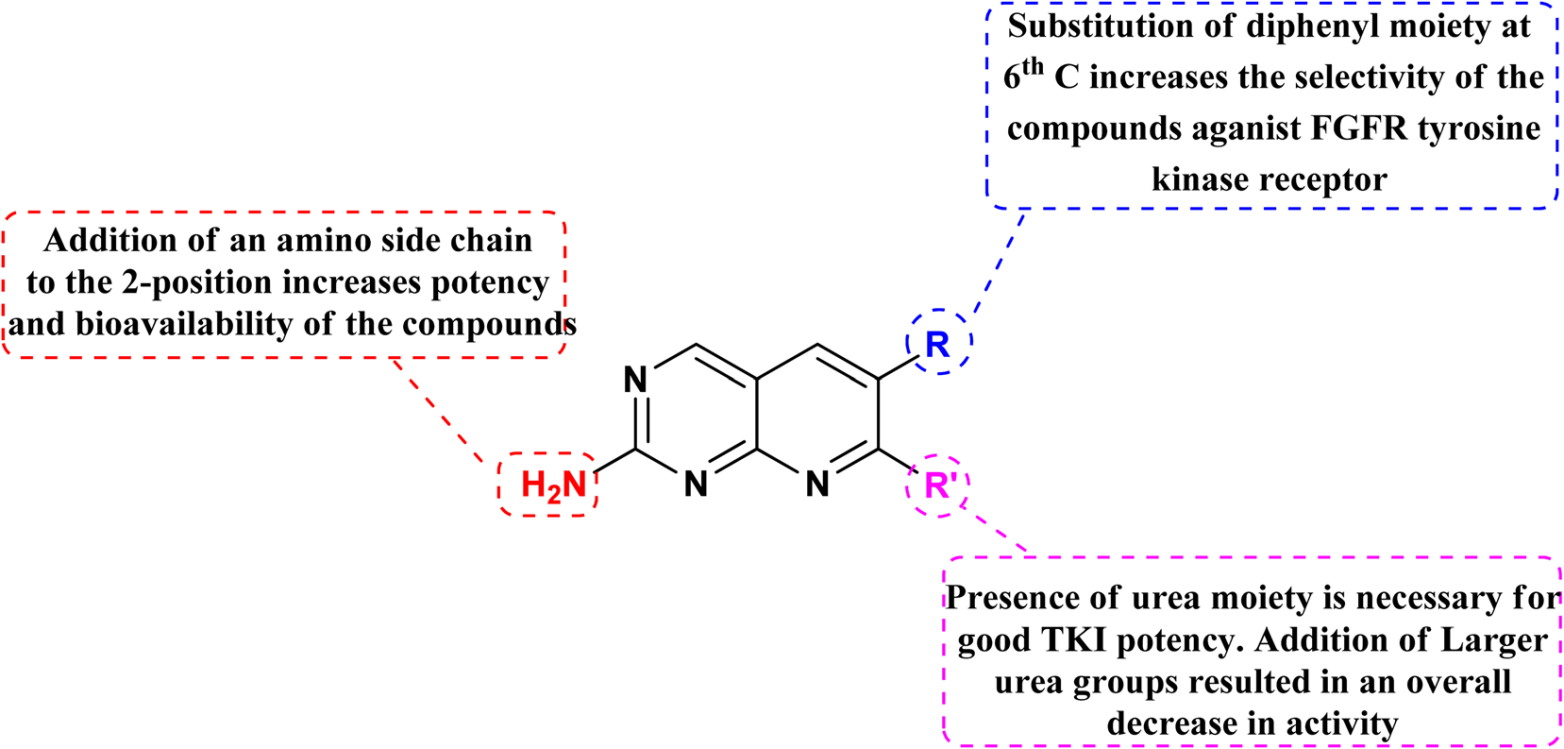
SAR of FGFR inhibitors.

**Fig. 41:**
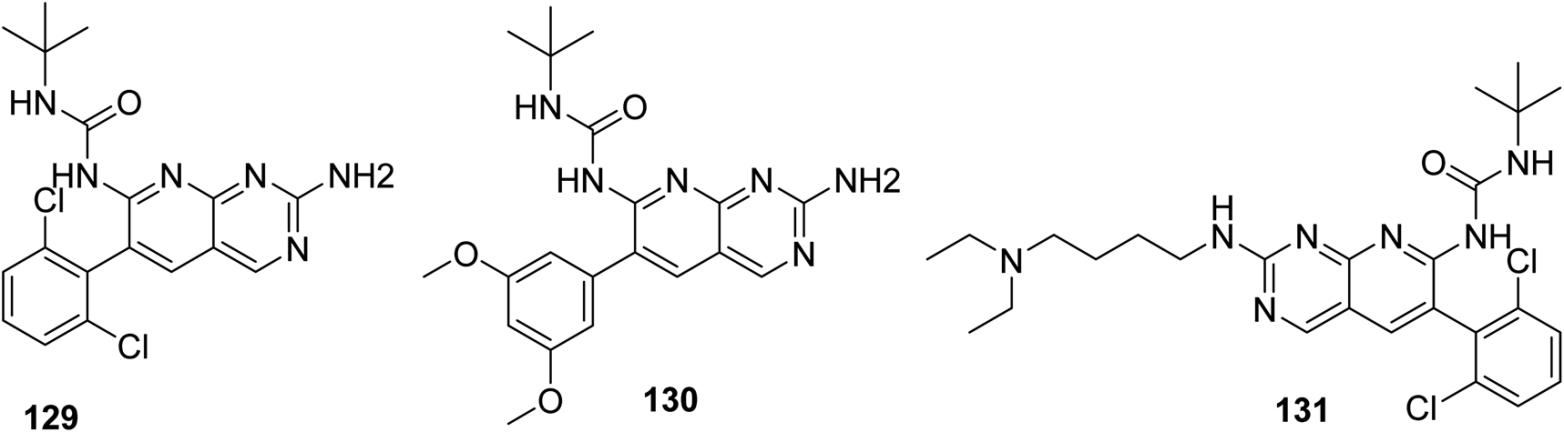
Structures of pyrido[2,3-*d*]pyrimidine derivatives as FGFR inhibitor.

SAR analysis revealed the creation of new analogs that were more potent, soluble, and bioavailable than the parent lead. Compound 129 was transformed into compound 131 [by introducing a {4-(diethylamino) butyl} amine substituent], which had a higher potency as well as availability with an IC_50_ of 0.3 μM and reduced PDGF-stimulated vascular smooth muscle cell growth. A selective FGFr tyrosine kinase antagonist, 130, was also created by substituting 6-(2,6-dichlorophenyl) group of 129 with the 6-(3′,5′-dimethoxyphenyl). If, the pyrido[2,3-*d*]pyrimidine nucleus has a N′-substituted alkylurea group on the seventh position of parent skeleton, excellent tyrosine kinase antagonists can be synthesiszed.^157^ Compound 131 inhibited the FGFr tyrosine kinase with an IC_50_ of 0.060 M, whereas the IC_50_s for the PDGFr, FGFr, EGFr, c-src, and InsR tyrosine kinases were all greater than 50 μM (Fig. 40 and 41).^157^

## 4 Summary of anticancer activity of pyrido[2,3-*d*]pyrimidine derivatives

**Fig. 42:**
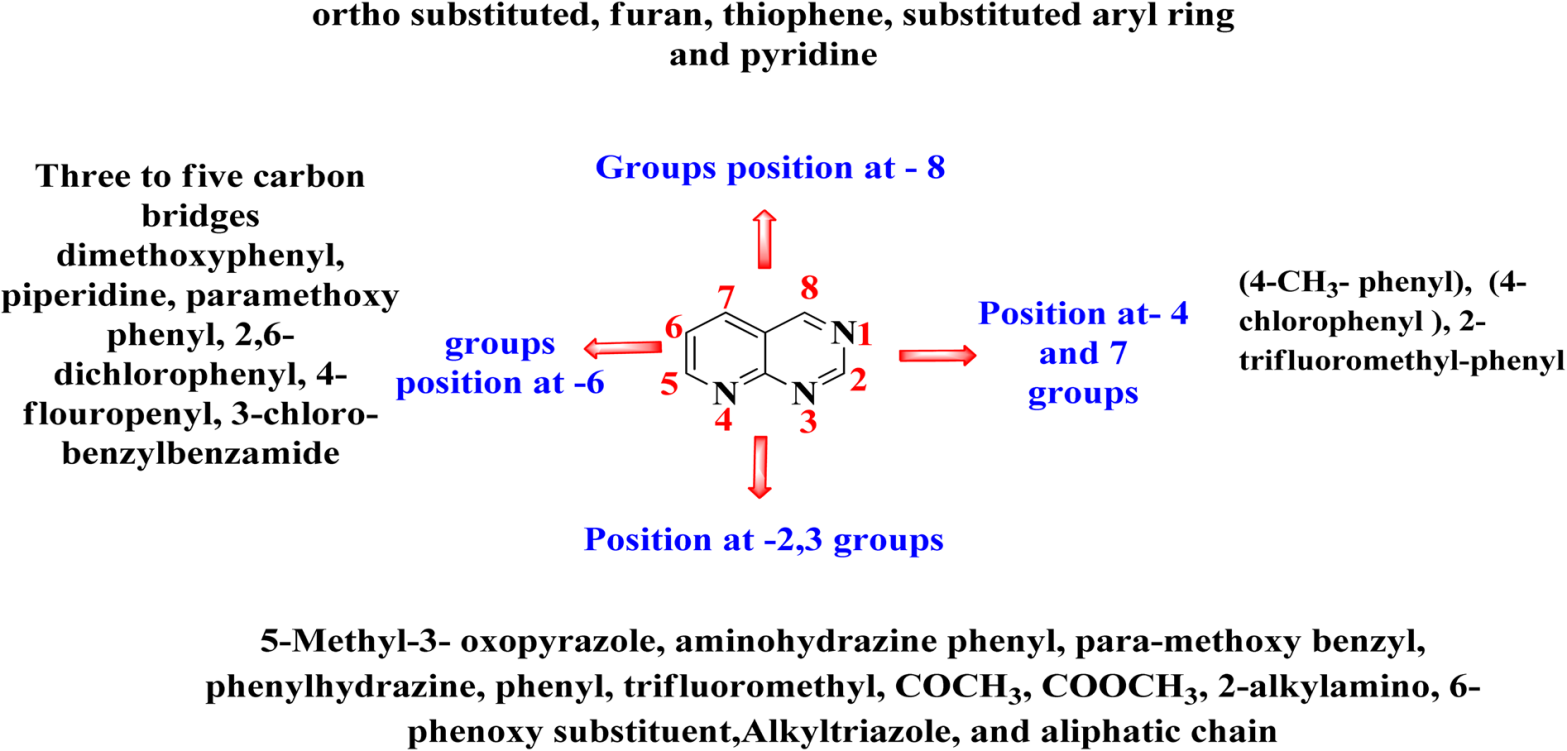
Summary of SAR of pyrido[2,3-*d*]pyrimidine derivatives as anticancer agents.

The overall SAR for the anticancer activity of pyrido[2,3-*d*]pyrimidine derivatives is shown below in Fig. 42.

## 5 Future perspective

Many FDA approved drugs like palbociclib, pamapimod, and piritrexim, having the pyrido[2,3-*d*]scaffold in their structure and are used to treat different form of cancer by *via* inhibiting CDK4/6, p38, and DHFR respectively. AMG510 and R1478 are under phase 1 clinical trails against KRAS and p38 respectively. Dilmapimod (100), TAK-733 (101), and compound 102 have been withdrawn from clinical trials due to undesirable side effects. Therefore, there is need to design selective molecules to combat resistance and problem of undesirable side effects. In this study, we have found many compounds showed excellent inhibition of cancer at the concentration of nM range. Researchers may design potent and selective molecules keeping in the consideration of stuructural requirements given in structure activity relationship study (Fig. 42).

## 6 Conclusion

Nitrogen containing heterocyclic compounds, especially pyrido[2,3-*d*]pyrimidines are a diverse class of chemicals having a number of biological properties. Tyrosine kinase, extracellular regulated protein kinases-ABL kinase, phosphatidylinositol-3 kinase, mammalian target of rapamycin, p38 mitogen-activated protein kinases, BCR-ABL, dihydrofolate reductase, Cyclin-dependent kinase, Phosphodiesterase, KRAS (the Gene Kirsten rat sarcoma viral oncogene homolog), and fibroblast growth factor receptors are the reported anticancer tagets of pyrido[2,3-*d*]pyrimidine. These derivatives have demonstrated significant anticancer action upon modifications at C-2, C-3, C-5, C-6, C-7, as well as C-8. Many researchers have explored its Structure activity relationship, as well as conformation and alignment parameters for ligand binding, employing computational modeling as well as docking investigations. The current study also emphasizes a variety of conventional, multi-component, and microwave-assisted approaches for synthesizing pyrido[2,3-*d*]pyrimidine derivatives and will also help scientists find highly effective, precise, and targeted pharmaceuticals.

## Conflicts of interest

The authors declare no competing interests.

## Author contributions

Conceptualization: P. K., and M. J.; data collection: A. K. S, and H. S.; writing the manuscript: A. K. and K. K. B; sketching of figures: A. K., T. A. and H. S.; data interpretation: A. H. E.; writing, review and final editing of the manuscript: A. V. and H. K.

## Supplementary Material
